# Mechanically induced oxidation of alcohols to aldehydes and ketones in ambient air: Revisiting TEMPO-assisted oxidations

**DOI:** 10.3762/bjoc.13.202

**Published:** 2017-10-02

**Authors:** Andrea Porcheddu, Evelina Colacino, Giancarlo Cravotto, Francesco Delogu, Lidia De Luca

**Affiliations:** 1 Dipartimento di Scienze Chimiche e Geologiche, Università degli Studi di Cagliari, Cittadella Universitaria, SS 554 bivio per Sestu, 09028 Monserrato (Ca), Italy; 2 Institut des Biomolécules Max Mousseron (IBMM) UMR5247 CNRS-UM-ENSCM, Université de Montpellier, Place Eugène Bataillon, cc1703, 34095 Montpellier Cedex 05, France; 3 Dipartimento di Scienza e Tecnologia del Farmaco, University of Turin, Via P. Giuria, 9, 10125 Turin, Italy; 4 Dipartimento di Ingegneria Meccanica, Chimica e dei Materiali, Università degli Studi di Cagliari, via Marengo 3, 09123 Cagliari, Italy; 5 Dipartimento di Chimica e Farmacia, Università degli Studi di Sassari, via Vienna 2, 07100 Sassari, Italy

**Keywords:** aldehydes, ball milling, ketones, mechanochemistry, oxidation reactions, TEMPO

## Abstract

The present work addresses the development of an eco-friendly and cost-efficient protocol for the oxidation of primary and secondary alcohols to the corresponding aldehydes and ketones by mechanical processing under air. Ball milling was shown to promote the quantitative conversion of a broad set of alcohols into carbonyl compounds with no trace of an over-oxidation to carboxylic acids. The mechanochemical reaction exhibited higher yields and rates than the classical, homogeneous, TEMPO-based oxidation.

## Introduction

Aldehydes and ketones constitute some of the most powerful and versatile building blocks that are available for a variety of synthetic transformations [1]. The reason for this lies in the capability of the carbonyl group to generate other possible functional groups through more or less complex chemical transformations [2]. The ubiquity of the carbonyl group in biomolecules adds further value to its chemistry, which is crucial for strategic areas of science related to biochemistry and biotechnology [3–4].

**Scheme 1:**
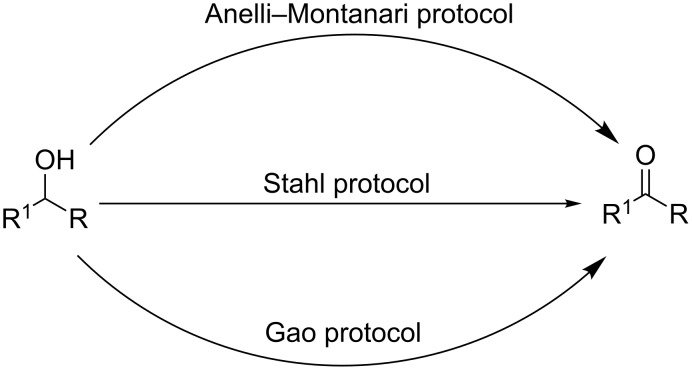
TEMPO-catalysed aerobic oxidative procedures of alcohols. a) Anelli–Montanari protocol: NaOCl (1.25 mol equiv), TEMPO (1–2 mol %), KBr (10 mol %), NaHCO_3_ (pH 8.6), CH_2_Cl_2_/H_2_O. b) Stahl protocol: [Cu(MeCN)_4_](OTf) (5 mol %), bpy (5 mol %), TEMPO (5 mol %), NMI (10 mol %), CH_3_CN, air. c) Gao (2016) protocol: Fe(NO)_3_^.^9H_2_O (10 mol %), 9-azabicyclo[3.3.1]nonan-*N*-oxyl (ABNO, 1–3 mol %), CH_3_CN, air.

In principle, the oxidation of alcohols represents a convenient option for preparing aldehydes and ketones, as alcohols are among the most abundant naturally occurring organic compounds [5–6]. Although the literature provides a plethora of generic indications and detailed recipes on this subject [7–10], the selective oxidation of primary alcohols to the corresponding aldehydes is one of the most difficult transformations to control because of the marked propensity towards over-oxidation to the respective carboxylic acid [11–12]. In addition, the appeal of this reaction is reduced by the need to use stoichiometric amounts of strong oxidising agents that are extremely toxic, hazardous, and expensive [13–17]. The use of the stable tetraalkylnitroxyl radical TEMPO (2,2,6,6-tetramethylpiperidine 1-oxyl) as the catalytic oxidising agent (Anelli–Montanari reaction) has been the main driving force behind the successful development of greener oxidation procedures [18–19]. The classic Anelli–Montanari oxidation requires aqueous NaOCl (bleach) as a co-oxidant, and it works in a CH_2_Cl_2_/H_2_O two-phase system buffered at pH 8.5–9.5 [20]. Over the years, bleach has been replaced with an impressively long list of other co-oxidants [21], which are sometimes very expensive, and exhibit a wide spectrum of effectiveness (Scheme 1) [22–23]. Recently, Stahl [24] developed a practical Cu^I^/TEMPO-based catalyst for the selective oxidation of primary alcohols to aldehydes under ambient aerobic conditions (Scheme 1) [25–26]. The procedure is operationally simple and extremely effective in terms of both chemoselectivity and reaction yield [27–28]. Gao (2016) further improved this methodology by replacing the bpy/Cu^I^/NMI catalyst system with Fe(NO_3_)_3_·9H_2_O, a cheaper, ligand-free co-oxidant (Scheme 1) [29–30]. This made the oxidative process more appealing for pharmaceutical applications, and specifically beneficial in the preparation of fragrances and food additives [31].

Despite the advances, the choice of solvent for TEMPO-based oxidative procedures remains a crucial issue in the development of greener alternatives to traditional alcohol oxidation reactions [32–34]. In particular, the lack of a green option significantly decreases the attractiveness of the proposed synthetic routes, as the solvent is the main component of the reaction system and, thus, the main source of waste in organic synthesis [35]. By far, performing the oxidation of alcohols under solvent-free conditions represents the best strategy to radically eliminate possible drawbacks in regard to waste disposal [36–37]. In this respect, the mechanical activation of solids [38–42], in the absence of solvents [43], or in the presence of catalytic amounts of liquid [44–45], holds significant promise [46–58].

Rooted in ancient practices from the dawn of civilization, a thin historical thread twisting across human history connects powder metallurgy and mineralurgy with science and engineering at the cutting edge of research in the fields of materials science and chemistry [59]. Presently, mechanochemistry is one of the fastest growing areas of investigation that aims to provide alternative methods to traditional syntheses in organic and inorganic chemistry [49,60–61]. Mechanochemistry is also used in supramolecular chemistry [62] and metal-organic chemistry [63].

In this work, we show that mechanical processing by ball milling can represent a viable solution to the selective oxidation of alcohols to aldehydes. Specifically, we investigated the potential of a mechanically activated TEMPO-based oxidative procedure [64].

## Results and Discussion

**Scheme 2:**
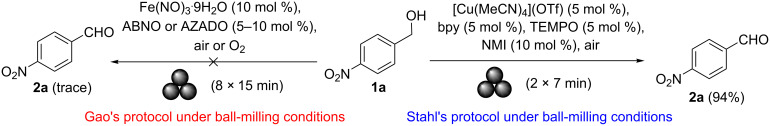
TEMPO-assisted oxidation of 4-nitrobenzylic alcohol under mechanical activation conditions [65].

We began our investigation with an attempt to replicate Gao’s procedure in a stainless steel reactor of a commercial ball mill in the presence of stainless steel balls and air, and in the absence of solvent. The oxidation of solid 4-nitrobenzyl alcohol (**1a**) to 4-nitrobenzaldehyde (**2a**) was selected as a model reaction. Unfortunately, the alcohol-to-aldehyde conversion was very low (<15%), and the use of larger amounts of the catalyst as well as molecular oxygen instead of air did not result in a significant improvement (Scheme 2, left side). To our great surprise, using Stahl’s catalyst, the mechanically activated oxidation of the model substrate **1a** under solvent-free conditions proceed so quickly and selectively that it was complete within just a few minutes. The progress of the reaction was monitored by TLC and GC–MS analysis until the completion of the reaction. The experimental protocol involved two stages, namely the preparation of the catalytic system and the final oxidation reaction. During the first stage [Cu(MeCN)_4_]OTf (5 mol %), 2,2′-bipyridine (5 mol %), NMI (10 mol %), and TEMPO (5 mol %) were milled (1 min) in a stainless steel reactor using four stainless steel balls of different sizes. Following the mechanical treatment, the catalyst uniformly covered the reactor walls forming a dark red/brown thin film. Subsequently, solid 4-nitrobenzyl alcohol (**1a**, 2 mmol) was added together with two more stainless steel balls (12 mm Ø), and the resulting mixture was milled until the starting alcohol was completely oxidized. Despite the poor reactivity of the 4-nitrobenzyl alcohol, the reaction smoothly reached completion in only 14 minutes (two cycles of 7 minutes each). GC–MS analysis of the crude reaction mixture only showed the presence of the desired aromatic aldehyde, indicating that over-oxidation did not occur (Scheme 2, right side). Prolonged milling did not result in the formation of detectable amounts of the carboxylic acid.

**Scheme 3:**
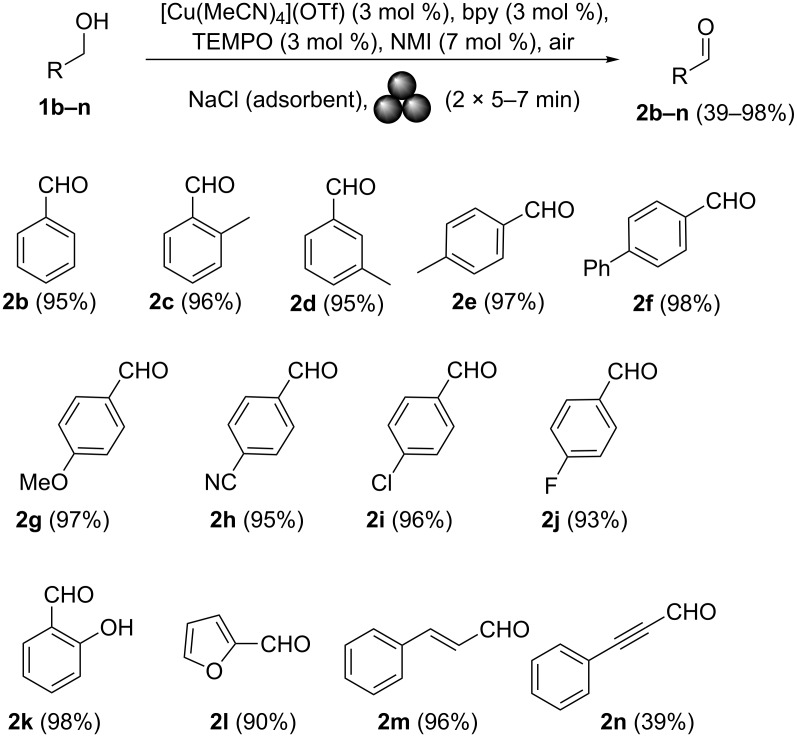
Scope of primary alcohols in oxidation under ambient air.

Next, we replaced the starting stainless-steel grinding jar and balls with a zirconia jar (45 mL) and six zirconium oxide balls (5 and 12 mm Ø) with the aim of avoiding contamination due to metal release. Under these conditions, it was possible to reduce the loading of [Cu(MeCN)_4_]OTf, 2,2′-bipyridine and TEMPO to 3 mol % and NMI loading to 7 mol % without affecting the reaction time or the product yield. Interestingly, the alcohol-to-aldehyde oxidation under ball milling conditions was faster (15 min overall) than that in solution (1 h) [25]. In addition, the absence of a solvent facilitated the purification of the final aldehyde. Specifically, the reaction crude was transferred from the reactor into a beaker containing an aqueous 10% citric acid solution [66–67], and the desired product precipitated as a solid. If necessary, the crude product could be further purified via filtering on a short pad of silica gel to give final aldehyde **2a** with a higher degree of purity (>95% as determined by GC–MS analysis). Since most common alcohols are, unfortunately, liquids at room temperature, their mechanical activation requires using a versatile dispersant. Ideally, a dispersant should not interfere with the oxidation reaction, and should be inexpensive and eco-friendly, if possible. As a first choice, we dispersed benzyl alcohol (**1b**) on alumina and silica gel. However, the reaction did not go to completion. In contrast, it proceeded smoothly (10 min) and in high yields when Na_2_SO_4_ and NaCl [68] were used as dispersants. Furthermore, the use of sodium chloride (500 mg per mmol of alcohol) facilitated the transfer of the reaction mixture from the reactor to the separating funnel containing the aqueous 10% citric acid solution. On the microscale (2 mmol), the full recovery of benzaldehyde was only achieved after solvent extraction. A minor modification to the synthetic protocol, involving the use of additional zirconia balls (four balls × 5 mm Ø, 7 balls × 12 mm Ø) and opening the jar (3 min) to air during the time interval between two consecutive cycles, gave **2b** in 96% overall yield even on the gram scale. On the gram scale, the mechanical activation no longer required an additional solvent to recover the final aldehyde during purification. With the optimized reaction conditions in hand, a series of common benzyl alcohols **1b–n** with different functional groups was then tested in order to examine the scope of the reaction (Scheme 3). To our satisfaction, very high yields (>90%) were obtained with all tested compounds, except **2n** (39%).

**Scheme 4:**
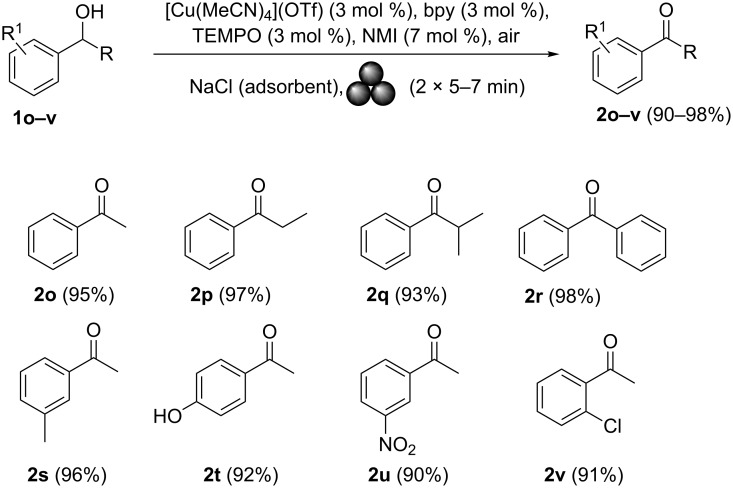
Scope of secondary alcohols in oxidation under ambient air.

Benzyl alcohols containing alkyl or aryl groups on the aromatic ring were all transformed into the desired products in quantitative or nearly quantitative isolated yields (compounds **2c–f** in Scheme 3). The position of the hydrocarbon (–R) on the ring did not significantly affect the aldehyde yield (aldehydes **2c–e** in Scheme 3). Substrates bearing electron-donating and electron-withdrawing functional groups on the aromatic ring of the benzyl alcohol were also viable, giving the corresponding aromatic aldehydes in high yields regardless of the electronic nature of their substituents (aldehydes **2g–k** in Scheme 3). Surprisingly, and contrary to Stahl’s original solution procedure [24], the oxidation of 2-hydroxybenzyl alcohol under mechanical activation conditions provided the salicylaldehyde in nearly quantitative yield (compound **2k** in Scheme 3). The reaction was also successfully expanded to heteroaromatic alcohol **1l** (Scheme 3, 2-furylmethanol), giving furfural in a very good yield (90%). The mechanically induced oxidative procedure was also applied to allylic alcohol derivatives. Cinnamyl alcohol (**1m**) was transformed into the corresponding α,β-unsaturated aldehyde in an excellent yield (96%) and with the stereochemical retention of the double bond. Encouraged by these promising results, we attempted to oxidise alkynols to the corresponding propargylic aldehyde derivatives, which were not previously accessible via classical homogeneous phase methods [25]. Contrary to our expectations, the ball milling protocol proved to be an efficient approach for the synthesis of these substrates, giving phenylpropargylaldehyde (**2n**) in a modest yield (39%) after 4 cycles (15 min per cycle). Unfortunately, prolonged milling times led to the decomposition of the final aldehyde. These promising results prompted us to undertake additional studies on secondary alcohols. The optimised ball milling protocol was applied to alcohols **1o–v**. Excellent yields of the ketones **2o–v** were obtained (Scheme 4). Notably, the product yield was not significantly affected by the position or electronic nature of the substituents on the aromatic ring of the alcohols.

Encouraged by the facile oxidation of benzyl alcohols, the scope of the reaction was finally extended to the formation of more challenging aliphatic aldehydes. Unfortunately, non-activated aliphatic alcohols did not react efficiently under the reaction conditions, and very low alcohol-to-aldehyde conversions occurred. The extension of milling times to 3 h failed to result in improved yields of all tested substrates: 3-phenyl-1-propanol, cyclohexanol and nonanol. Despite several attempts to improve the alcohol-to-aldehyde conversion, by, for instance, milling under an oxygen atmosphere and the use of more reactive co-oxidant catalysts [69], no significant improvements were observed.

## Conclusion

We have developed a TEMPO-based oxidative procedure for the air oxidation of primary and secondary benzyl alcohols to the corresponding aldehydes and ketones under ball milling conditions. A library of common alcohols was efficiently converted into carbonyl compounds with no trace of over-oxidation to the carboxylic acids. The final products could be easily separated/purified from the crude reaction mixture without using toxic organic solvents. Under mechanical activation conditions, the reactions provided better yields and proceeded faster than classical, homogeneous phase TEMPO-based oxidations. Studies are underway to identify more effective TEMPO-based catalysts that are also capable of promoting the oxidation of non-activated alcohols.

## Experimental

General procedure to prepare carbonyl compounds **2a–v**. 2,2,6,6-Tetramethylpiperidine 1-oxyl (TEMPO, 9.4 mg, 0.06 mmol, 3 mol %), 2,2′-bipyridyl (9,4 mg, 0.06 mmol, 3 mol %), [Cu(CN)_4_]OTf (22.6 mg, 0.06 mmol, 3 mol %) and 1-methylimidazole (NMI, 11.5 mg, 11.2 μL, 0.14 mmol, 7 mol %) were placed in a zirconia-milling beaker (45 mL) equipped with four balls (two balls × 5 mm Ø, two balls × 12 mm Ø) of the same material. The jar was sealed and ball-milled for 1 min. Then, benzyl alcohol (216.3 mg, 207 μL, 2.0 mmol), NaCl (1.0 g) together with other two zirconia balls (12 mm Ø) were added and the reaction mixture was subjected to grinding for further 10 minutes overall (two cycles of 5 minutes each). The first milling cycle was followed by a break of 2 min leaving in the meantime the uncovered jar in open air. The progress of the reaction was monitored by TLC analysis (heptane/AcOEt 9:1 v/v) and GC–MS analysis on an aliquot of the crude. Upon completion of the ball milling process, the jar was opened, the milling balls were removed and the resulting crude product (adsorbed on NaCl) was then easily transferred into a separating funnel filled with an aqueous 10% citric acid solution (20 mL). The aqueous phase was extracted with cyclopentyl methyl ether (or alternatively with AcOEt) (3 × 15 mL). The combined organic fractions were washed with H_2_O (25 mL) and brine (25 mL), then dried over Na_2_SO_4_, and concentrated in vacuo to give benzaldehyde in high yield (195 mg, 92%) and good purity (>93% by GC analysis). Alternatively, after completion of the reaction, the resulting crude product (adsorbed on NaCl) can be also easily purified by a short column chromatography on silica gel using heptane/ethyl acetate (9:1 v/v) as the eluents to afford pure aldehyde **2b** in high yield (202 mg, 95%) as a colourless liquid.

## Supporting Information

File 1 Experimental part and NMR spectra.
