# Supplementary material for: Mechanically induced oxidation of alcohols to aldehydes and ketones in ambient air: Revisiting TEMPO-assisted oxidations
Source: Beilstein J Org Chem. 2017 Oct 2;13:2049–55. doi: 10.3762/bjoc.13.202 (PMC5647725; doi:10.3762/bjoc.13.202)
Supplement: File 1 — Experimental part and NMR spectra. [file Beilstein_J_Org_Chem-13-2049-s001.pdf]

Supporting Information

for

Mechanically induced oxidation of alcohols to aldehydes and ketones in ambient air: Revisiting TEMPO-assisted oxidations

Andrea Porcheddu*[1], Evelina Colacino[1,2], Giancarlo Cravotto[3], Francesco Delogu[4], and Lidia De Luca[5]

Address: [1]Dipartimento di Scienze Chimiche e Geologiche, Università degli Studi di Cagliari, Cittadella Universitaria, SS 554 bivio per Sestu, 09028 Monserrato (Ca), Italy, [2]Institut des Biomolécules Max Mousseron (IBMM) UMR5247 CNRS-UM-ENSCM, Université de Montpellier, Place Eugène Bataillon, cc1703, 34095 Montpellier Cedex 05, France, [3]Dipartimento di Scienza e Tecnologia del Farmaco, University of Turin, Via P. Giuria, 9 - 10125 Turin, Italy, [4]Dipartimento di Ingegneria Meccanica, Chimica e dei Materiali, Università degli Studi di Cagliari, via Marengo 3, 09123 Cagliari, Italy and [5]Dipartimento di Chimica e Farmacia, Università degli Studi di Sassari, via Vienna 2, 07100 Sassari, Italy.

Emai: Andrea Porcheddu - porcheddu@unica.it

*Corresponding author

Experimental part and NMR spectra

TABLE OF CONTENTS

Section	Page
Table of contents	S2
1. General methods and materials	S3
2. General procedures for the oxidation of benzyl alcohols	S4
3. Summary of ^{1}H and ^{13}C NMR data for carbonyl compounds **2a**–**v**	S5
4. ^{1}H and ^{13}C NMR spectra for carbonyl compounds **2a**–**v**	S10

1. General methods and materials

Commercially available reagents were purchased from Acros, Aldrich, Strem Chemicals, Alfa-Aesar, TCI Europe and were used as received. The solvents were purchased from Aldrich or VWR International in sure/sealedTM bottles over molecular sieves. Flash column chromatography was performed with EcoChrome^{TM} MP Silica gel 60A, particle size 0.040–0.063 mm (230–400 mesh). All reactions were monitored by thin-layer chromatography (TLC) performed on glass-backed silica gel 60 F254, 0.2 mm plates (Merck), and compounds were visualized under UV light (254 nm) or using cerium ammonium molybdate solution with subsequent heating. The eluents were technical grade and distilled prior to use. A Spex 8000M Mixer/Mill® apparatus was used for all reactions (875 rpm, 14.6 Hz). ^{1}H and ^{13}C liquid NMR spectra were recorded on Varian 400 and 500 MHz NMR spectrometers at 25 °C and are calibrated using tetramethylsylane (TMS). Proton chemical shifts are expressed in parts per million (ppm, δ scale) and are referred to the residual hydrogen in the solvent ($CHCl_3$, 7.27 ppm or DMSO-d_6 2.54 ppm). Data are represented as follows: chemical shift, multiplicity (s = singlet, d = doublet, t = triplet, q = quartet, m = multiplet and/or multiple resonances, br s = broad singlet), coupling constant (J) in Hertz and integration. Carbon chemical shifts are expressed in parts per million (ppm, δ scale) and are referenced to the carbon resonances of the NMR solvent ($CDCl_3$, δ 77.0 ppm or δ DMSO-d_6 δ 39.5 ppm). Deuterated NMR solvents were obtained from Aldrich. High-resolution mass spectra (HRMS) were recorded using an Electrospray Ionisation (ESI) spectrometer. Analysis of reaction mixture was determined by GC–MS (GC Agilent 6850, MS Agilent 5973) and equipped with HP5 universal capillary column (30 m length and 0.20 mm diameter, 0.11 film thickness) and a flame ionization detector (FID). GC oven temperature was programmed from 80 °C to 250 °C at the rate of 10 °C/min. He gas was used as a carrier gas. Temperatures of injection port and FID were kept constant at 300 °C. Retention times of different compounds were determined by injecting the pure compound under identical conditions, respectively. Melting points were determined in an open capillary on a Büchi melting point apparatus and are uncorrected. All the experiments were carried out in duplicate to ensure reproducibility of the experimental data. Yields refer to pure isolated materials. Spectroscopic data were in agreement with those of the commercially available reference compounds.

2. General procedures for the oxidation of benzyl alcohols

General procedure to prepare carbonyl compounds **2a–v**. 2,2,6,6-Tetramethylpiperidine 1-oxyl (TEMPO, 9.4 mg, 0.06 mmol, 3 mol %), 2,2′-bipyridyl (9,4 mg, 0.06 mmol, 3 mol %), $[Cu(CN)_4]OTf$ (22.6 mg, 0.06 mmol, 3 mol %) and 1-methylimidazole (NMI, 11.5 mg, 11.2 μL, 0.14 mmol, 7 mol%) were placed in a zirconia-milling beaker (45 mL) equipped with four balls (two balls × 5 mm Ø, two balls × 12 mm Ø) of the same material. The jar was sealed and ball-milled for 1 min. Then, benzyl alcohol (216.3 mg, 207 μL, 2.0 mmol), NaCl (1.0 g) together with other two zirconia balls (12 mm Ø) were added and the reaction mixture was subjected to grinding further for 10 minutes overall (two cycles of 5 minutes each). The first milling cycle was followed by a break of 2 min leaving in the meantime the uncovered jar in open air. The progress of the reaction was monitored by TLC analysis (heptane/AcOEt: 9/1 v/v) and GC–MS analysis on an aliquot of crude. Upon completion of the ball milling process, the jar was opened, the milling balls were removed and the resulting crude product (adsorbed on NaCl) was then easily transferred into a separating funnel filled with an aqueous 10% citric acid solution (20 mL). The aqueous phase was extracted with cyclopentyl methyl ether (or alternatively with AcOEt) (3 × 15 mL). The combined organic fractions were washed with H_2O (25 mL) and brine (25 mL), then dried over Na_2SO_4, and concentrated in vacuo to give benzaldehyde in high yield (195 mg, 92%) and good purity (>93% by GC analysis). Alternatively, after completion of the reaction, the resulting crude product (adsorbed on NaCl) can be also easily purified by a short column chromatography on silica gel using heptane/ethyl acetate (9/1 v/v) as the eluents to afford pure aldehyde **2b** in high yield (202 mg, 95%) as a colourless liquid.

3. Summary of ^{1}H and ^{13}C NMR data for carbonyl compounds 2a–v

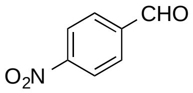

- 4-Nitrobenzaldehyde**, 2a** (284 mg, 94%), CAS Number 555-16-8.

^{1}H NMR (500 MHz, DMSO-d_6) δ 10.17 (s, 1H), 8.42 (d, *J* = 8.6 Hz, 2H), 8.17 (d, *J* = 8.6 Hz, 2H). **^{13}C NMR** (126 MHz, DMSO-d_6) δ 192.3, 150.6, 140.0, 130.6, 124.3.

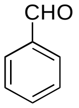

- Benzaldehyde, **2b** (202 mg, 95%), CAS Number 100-52-7.

^{1}H NMR (500 MHz, $CDCl_3$) δ 10.03 (s, 1H), 7.90 (d, *J* = 6.7 Hz, 2H), 7.67–7.61 (m, 1H), 7.58–7.50 (m, 2H). **^{13}C NMR** (126 MHz, $CDCl_3$) δ 192.4, 136.5, 134.5, 129.8, 129.1.

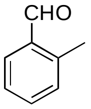

- 2-Methylbenzaldehyde, **2c** (231 mg, 96%) CAS Number 529-20-4.

^{1}H NMR (500 MHz, $CDCl_3$) δ 10.29 (s, 1H), 7.81 (d, *J* = 7.6 Hz, 1H), 7.49 (t, *J* = 7.6 Hz, 1H), 7.38 (t, *J* = 7.6 Hz, 1H), 7.29 (d, *J* = 7.6 Hz, 1H), 2.69 (s, 3H). **^{13}C NMR** (126 MHz, $CDCl_3$) δ 195.3, 143.2, 136.8, 136.2, 134.6, 134.4, 128.9, 22.1.

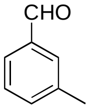

- 3-Methylbenzaldehyde, **2d** (228 mg, 95 %), CAS Number 620-23-5.

^{1}H NMR (500 MHz, $CDCl_3$) δ 10.01 (s, 1H), 7.72-7.69 (m, 2H), 7.50–7.41 (m, 2H), 2.46 (s, 3H). **^{13}C NMR** (126 MHz, $CDCl_3$) δ 192.1, 138.5, 136.2, 134.9, 129.6, 128.5, 126.8, 20.8.

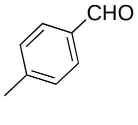

- 4-Methylbenzaldehyde, **2e** (233 mg, 97%), CAS Number 104-87-0.

^{1}H NMR (500 MHz, $CDCl_3$) δ 9.99 (s, 1H), 7.80 (d, *J* = 8.0 Hz, 2H), 7.35 (d, *J* = 8.0 Hz, 2H), 2.46 (s, 3H). **^{13}C NMR** (126 MHz, $CDCl_3$) δ 194.5, 148.1, 136.9, 132.4, 132.3, 24.5.

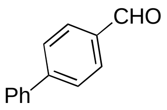

- [1,1'-Biphenyl]-4-carbaldehyde, **2f** (357 mg, 98%), CAS Number 3218-36-8.

^{1}H NMR (500 MHz, $CDCl_3$) δ 10.08 (s, 1H), 7.97 (d, *J* = 8.4 Hz, 2H), 7.77 (d, *J* = 8.4 Hz, 2H), 7.66 (d, *J* = 7.3 Hz, 2H), 7.51 (t, *J* = 7.3 Hz, 2H) 7.45 (t, *J* = 7.3 Hz, 1H). **^{13}C NMR** (126 MHz, $CDCl_3$) δ 192.1, 147.3, 139.9, 135.5, 130.5, 129.3, 128.8, 127.9, 127.6.

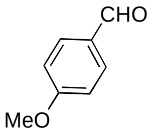

- 4-Methoxybenzaldehyde, **2g** (264 mg, 97%), CAS Number 123-11-5.

^{1}H NMR (500 MHz, $CDCl_3$) δ 9.89 (s, 1H), 7.84 (d, *J* = 8.7 Hz, 2H), 7.01 (d, *J* = 8.7 Hz, 2H), 3.89 (s, 3H). **^{13}C NMR** (126 MHz, $CDCl_3$) δ 193.4, 167.3, 134.6, 132.6, 116.9, 58.2.

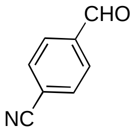

- 4-Formylbenzonitrile, **2h** (249 mg, 95%), CAS Number 105-07-7.

^{1}H NMR (500 MHz, $CDCl_3$) δ 10.11 (s, 1H), 8.01 (d, *J* = 8.6 Hz, 2H), 7.86 (d, *J* = 8.6 Hz, 2H). **^{13}C NMR** (126 MHz, $CDCl_3$) δ 190.4, 138.6, 132.8, 129.7, 117.5, 107.2.

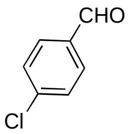

- 4-Chlorobenzaldehyde, **2i** (270 mg, 96%), CAS Number 104-88-1.

^{1}H NMR (500 MHz, $CDCl_3$) δ 10.00 (s, 1H), 7.84 (d, *J* = 8.4 Hz, 2H), 7.53 (d, *J* = 8.4 Hz, 2H). **^{13}C NMR** (126 MHz, $CDCl_3$) δ 190.9, 141.1, 134.9, 131.0, 129.6.

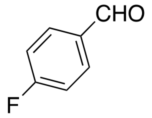

- 4-Fluorobenzaldehyde, **2j** (231 mg, 93%), CAS Number 459-57-4

^{1}H NMR (500 MHz, $CDCl_3$) δ 9.98 (s, 1H), 7.94-7.91 (m, 2H), 7.22 (t, J = 8.4 Hz, 2H). **^{13}C NMR** (126 MHz, $CDCl_3$) δ 190.6, 166.6 (d, $^{1}J_{C\text{-}F}$ = 255 Hz), 133.1 (d, $^{4}J_{C\text{-}F}$ = 3.0 Hz), 132.4 (d, $^{3}J_{C\text{-}F}$ = 10 Hz), 116.5 (d, $^{2}J_{C\text{-}F}$ = 23 Hz).

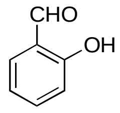

- 2-Hydroxybenzaldehyde, **2k** (239 mg, 98%), CAS Number 90-02-8.

^{1}H NMR (500 MHz, $CDCl_3$) δ 11.03 (bs, 1H), 9.93 (s, 1H), 7.61–7.52 (m, 2H), 7.06–6.99 (m, 2H). **^{13}C NMR** (126 MHz, $CDCl_3$) δ 199.2, 164.3, 139.6, 136.4, 123.3, 122.5, 120.2.

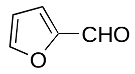

- Furan-2-ylcarbaldehyde, **2l** (173 mg, 90%, hexane/AcOEt 95/5), CAS Number 98-01-1.

^{1}H NMR (500 MHz, $CDCl_3$) δ 9.69 (s, 1H), 7.71 (d, J = 1.6 Hz, 1H), 7.27 (d, J = 3.5 Hz, 1H), 6.62 (dd, ^{1}J = 3.5 Hz, ^{2}J= 1.6 Hz, 1H). **^{13}C NMR** (126 MHz, $CDCl_3$) δ 178.0, 153.2, 148.2, 123.4 112.7.

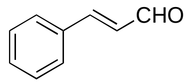

- *Trans*-cinnamaldehyde, **2m** (254 mg, 96%), CAS Number 14371-10-9.

^{1}H NMR (500 MHz, $CDCl_3$) δ 9.74 (d, J = 7.7 Hz, 1H), 7.62–7.57 (m, 2H), 7.54–7.44 (m, 4H), 6.75 (dd, J = 16.0, 7.7 Hz, 1H). **^{13}C NMR** (126 MHz, $CDCl_3$) δ 193.9, 152.9, 134.3, 131.5, 129.4, 128.9, 128.7.

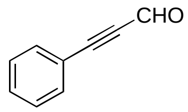

- 3-Phenylpropiolaldehyde, **2n** (101 mg, 39%), CAS Number 2579-22-8.

^{1}**H NMR** (500 MHz, Chloroform-*d*) δ 9.44 (s, 1H), 7.62 (d, *J* = 9.4 Hz, 2H), 7.51 (t, *J* = 7.7 Hz, 1H), 7.42 (t, *J* = 7.7 Hz, 2H). ^{13}**C NMR** (126 MHz, $CDCl_3$) δ 176.8, 133.3, 131.3, 128.7, 119.4, 95.1, 88.4.

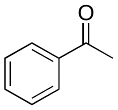

- Acetophenone, **2o** (228 mg, 95%), CAS Number 98-86-2.

^{1}**H NMR** (500 MHz, $CDCl_3$) δ 8.01–7.95 (m, 2H), 7.62–7.55 (m, 1H), 7.52–7.45 (m, 2H), 2.63 (s, 3H). ^{13}**C NMR** (126 MHz, $CDCl_3$) δ 198.2, 137.3, 133.2, 128.7, 128.4, 26.7.

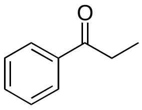

- Propiophenone, **2p** (260 mg, 97%), CAS Number 93-55-0.

^{1}**H NMR** (500 MHz, $CDCl_3$) δ 8.02–7.96 (m, 2H), 7.61–7.54 (m, 1H), 7.51–7.44 (m, 2H), 3.03 (q, *J* = 7.2 Hz, 2H), 1.25 (t, *J* = 7.2 Hz, 3H). ^{13}**C NMR** (126 MHz, $CDCl_3$) δ 203.4, 139.6, 135.5, 131.2, 130.6, 34.4, 10.9.

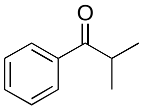

- 2-Methyl-1-phenylpropan-1-one, **2q** (276 mg, 93%), CAS Number 611-70-1.

^{1}**H NMR** (500 MHz, $CDCl_3$) δ 7.96 (d, *J* = 7.9 Hz, 2H), 7.57–7.50 (m, 1H), 7.48–7.41 (m, 2H), 3.55 (hept, *J* = 6.8 Hz, 1H), 1.21 (d, *J* = 6.8 Hz, 6H). ^{13}**C NMR** (126 MHz, $CDCl_3$) δ 204.7, 136.5, 133.0, 128.8, 128.6, 35.6, 19.4.

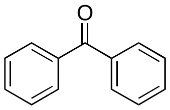

- Benzophenone, **2r** (357 mg, 98%), CAS Number 119-61-9.

^{1}**H NMR** (500 MHz, $CDCl_3$) δ 7.86–7.80 (m, 2H), 7.60 (td, *J* = 7.8, 1.2 Hz, 1H), 7.53–7.46 (m, 2H). ^{13}**C NMR** (126 MHz, $CDCl_3$) δ 197.0, 137.9, 132.7, 130.4, 128.6.

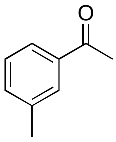

- 1-(*m*-Tolyl)ethanone, **2s** (258 mg, 96%), CAS Number 585-74-0.

^{1}H NMR (500 MHz, $CDCl_3$) δ 7.78–7.75 (m, 2H), 7.37–7.33 (m, 2H), 2.59 (s, 3H), 2.41 (s, 3H). ^{13}C NMR (126 MHz, $CDCl_3$) δ 198.4, 138.4, 137.2, 133.9, 128.9, 128.5, 125.6, 26.7, 21.4.

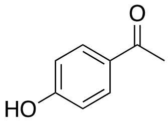

- 1-(4-Hydroxyphenyl)ethan-1-one, **2t** (250 mg, 92%), CAS Number 99-93-4.

^{1}H NMR (500 MHz, $CDCl_3$) δ 8.09 (s, 1H), 7.94 (d, *J* = 8.7 Hz, 2H), 6.98 (d, *J* = 8.7, 2H), 2.61 (s, 3H). **^{13}C NMR** (126 MHz, $CDCl_3$) δ 199.0, 161.8, 131.4, 129.6, 115.6, 26.4.

- 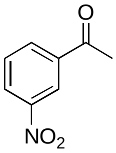

- 1-(3-Nitrophenyl)ethanone, **2u** (297 mg, 90%), CAS Number 121-89-1.

^{1}H NMR (500 MHz, $CDCl_3$) δ 8.77 (d, *J* = 2.0 Hz, 1H), 8.44–8.39 (m, 1H), 8.32–8.26 (m, 1H), 7.70 (t, *J* = 7.8 Hz, 1H), 2.70 (s, 3H). **^{13}C NMR** (126 MHz, $CDCl_3$) δ 195.9, 148.7, 138.6, 134.1, 130.2, 127.7, 123.5, 27.0.

- 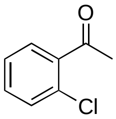

- 1-(2-Chlorophenyl)ethanone, **2v** (281 mg, 91%), CAS Number 99-02-5.

^{1}H NMR (500 MHz, $CDCl_3$) δ 7.59–7.53 (m, 1H), 7.49–7.36 (m, 2H), 7.36–7.26 (m, 1H), 2.66 (s, 3H). **^{13}C NMR** (126 MHz, $CDCl_3$) δ 200.7, 139.5, 132.3, 131.7, 131.0, 129.7, 127.3, 31.1.

4. ^{1}H and ^{13}C NMR spectra for carbonyl compounds 2a–v

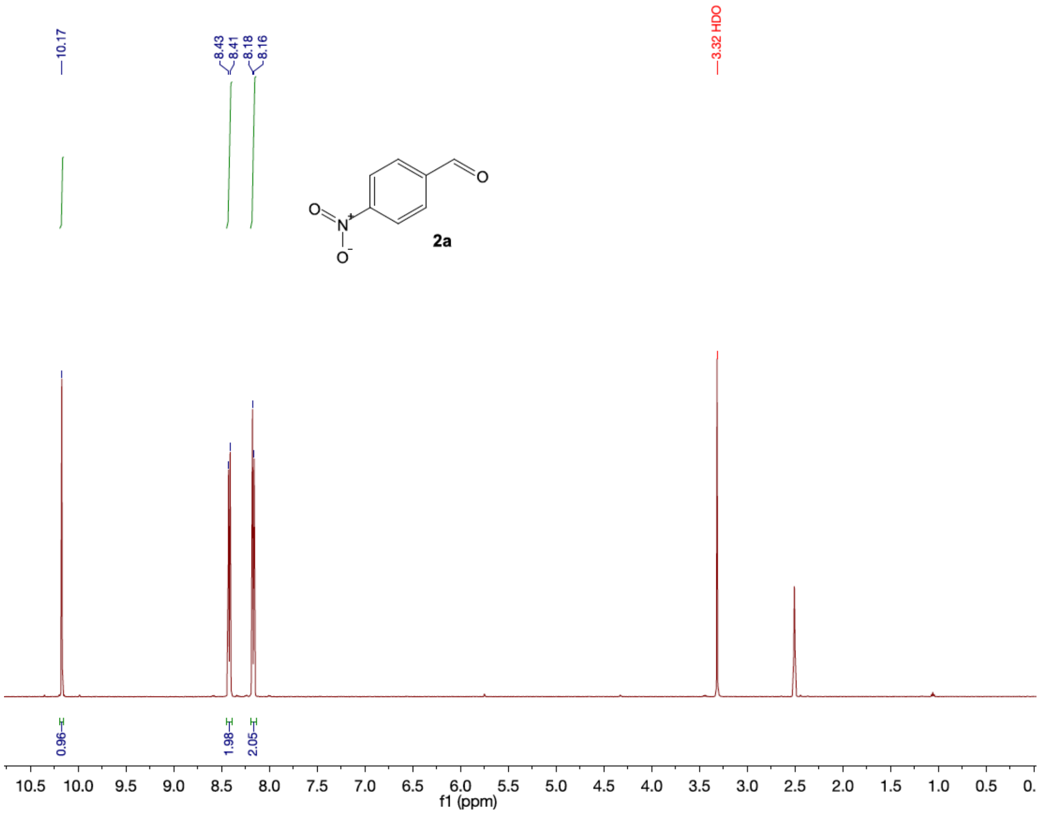

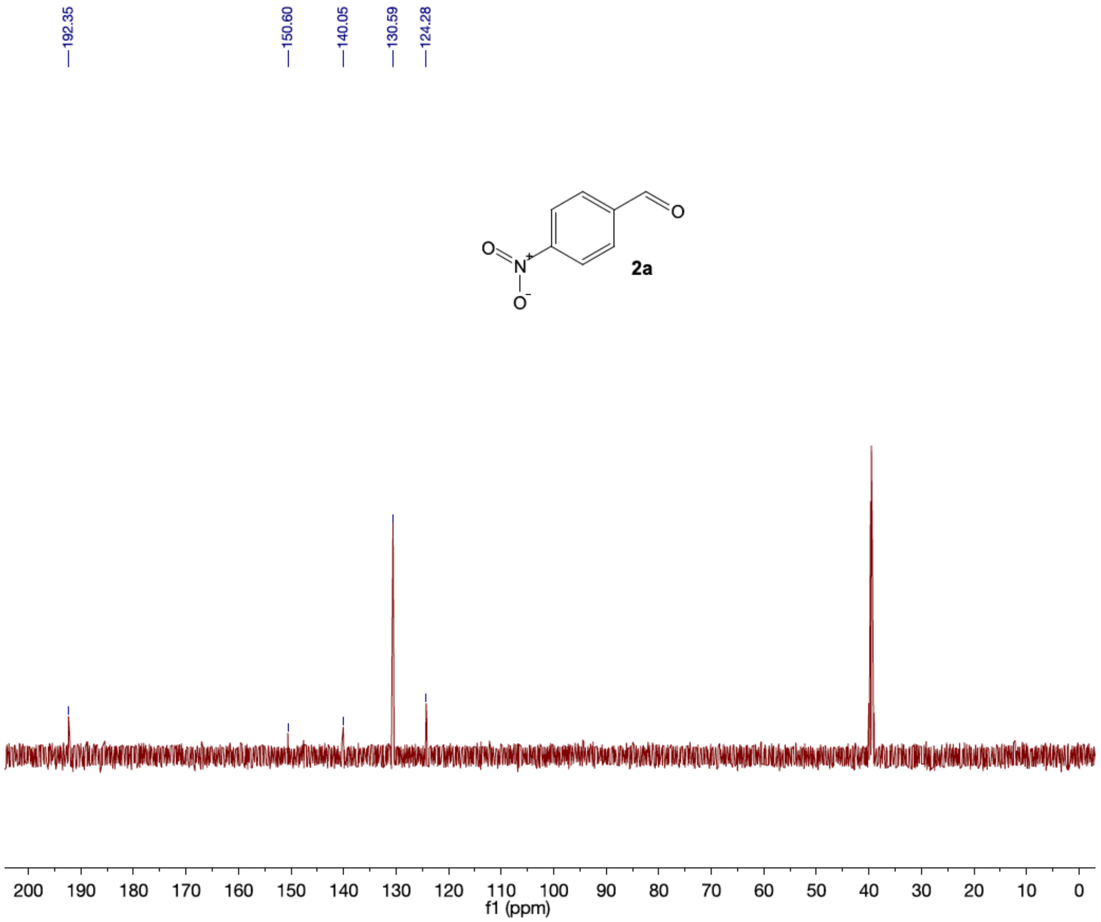

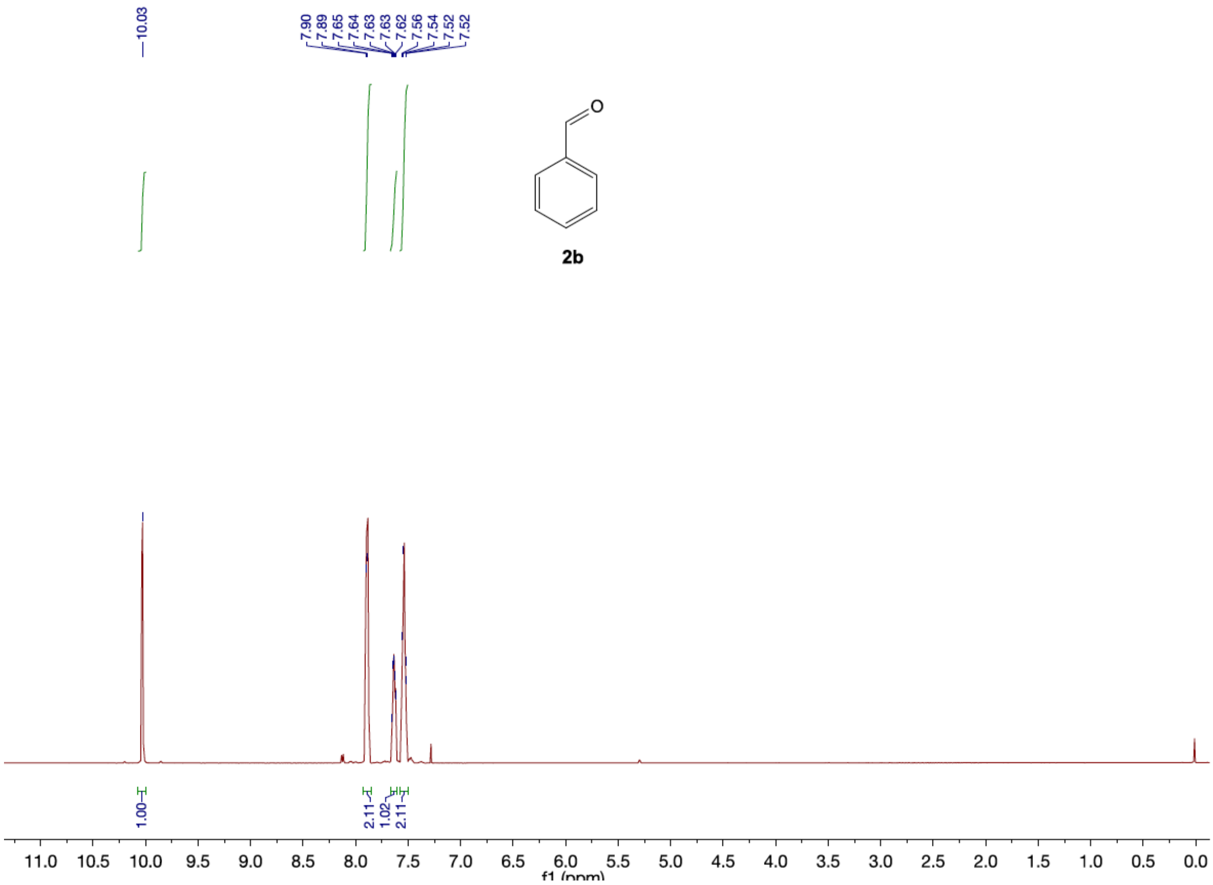
10.03
7.90
7.89
7.65
7.64
7.63
7.63
7.62
7.56
7.54
7.52
7.52
1.00
2.11
1.02
2.11
11.0 10.5 10.0 9.5 9.0 8.5 8.0 7.5 7.0 6.5 6.0 5.5 5.0 4.5 4.0 3.5 3.0 2.5 2.0 1.5 1.0 0.5 0.0
f1 (ppm)

2b

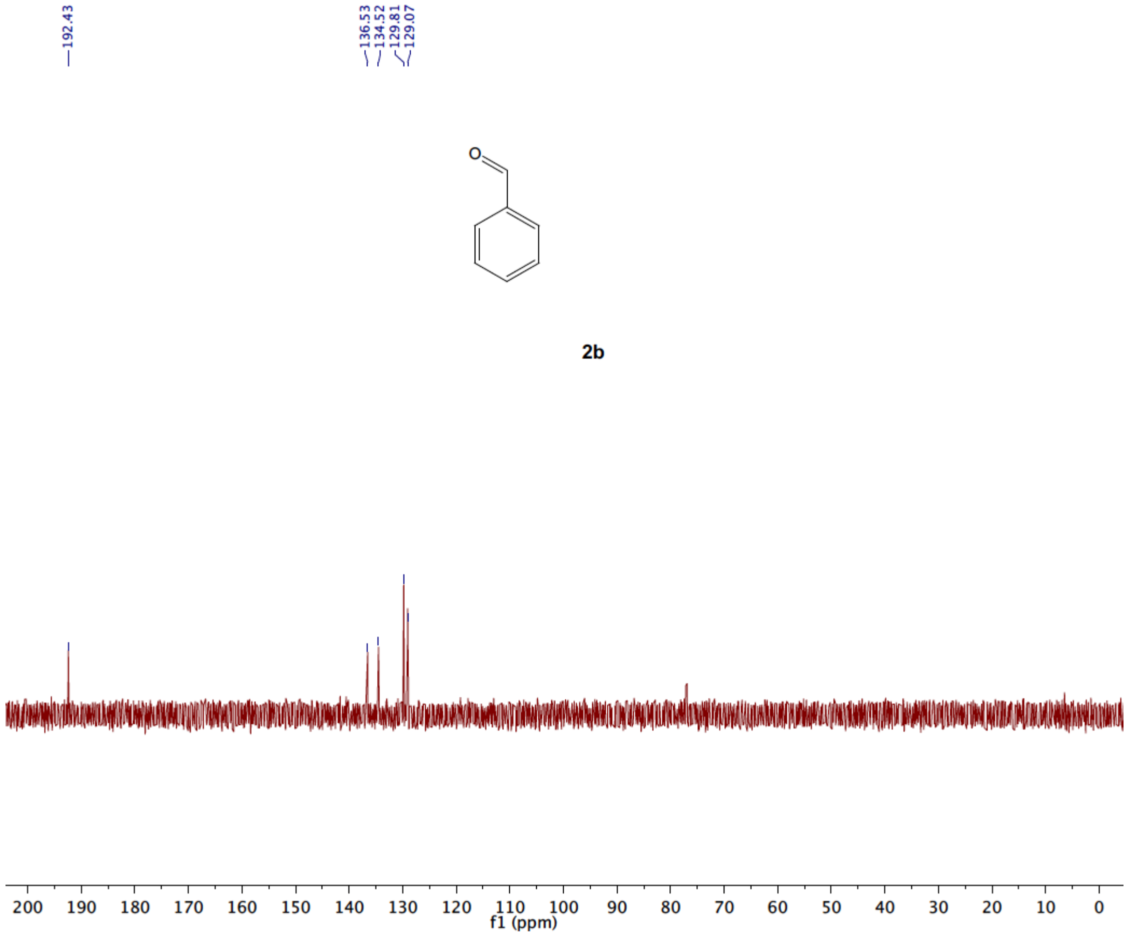
192.43
136.53
134.52
129.81
129.07
200 190 180 170 160 150 140 130 120 110 100 90 80 70 60 50 40 30 20 10 0
f1 (ppm)

2b

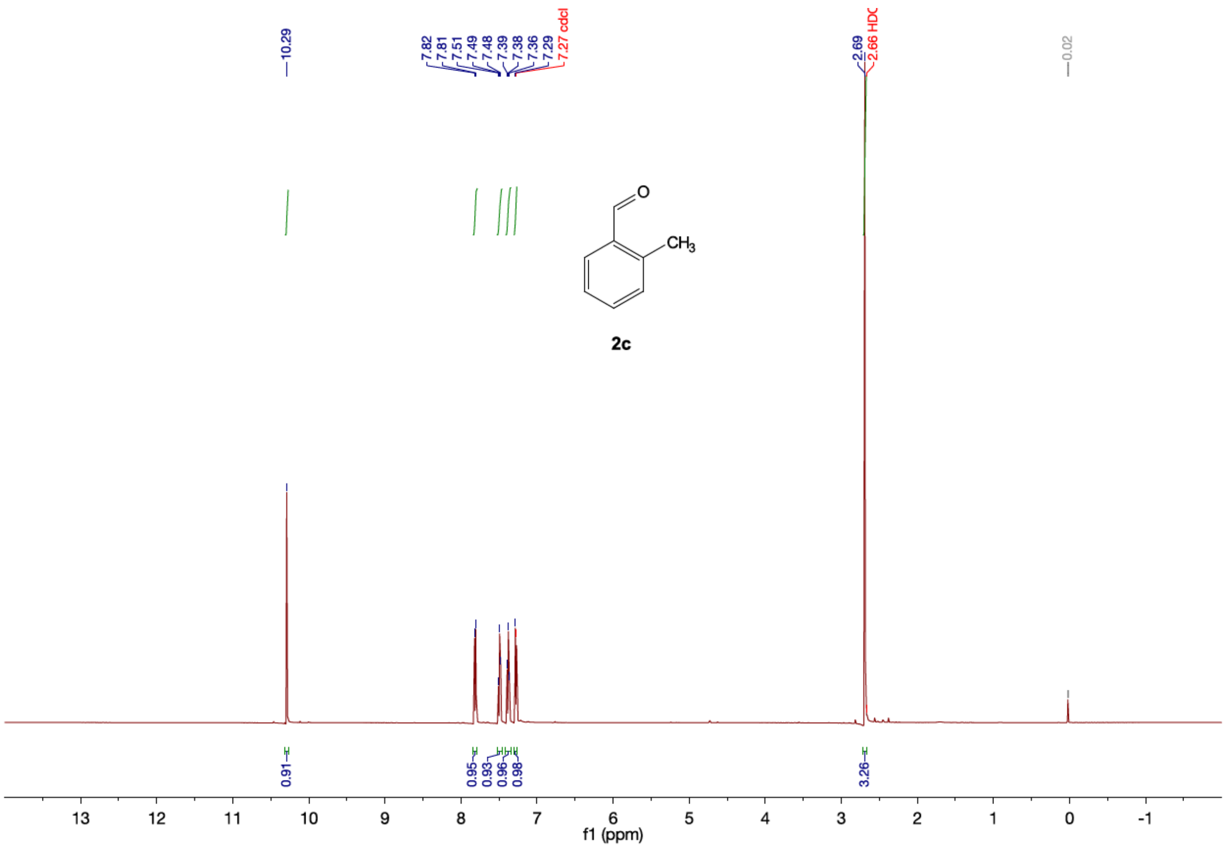
10.29
7.82
7.81
7.51
7.49
7.48
7.39
7.38
7.36
7.29
7.27 cdcl
2.69
2.66 HDO
0.02
O
CH3
2c
0.91
0.95
0.93
0.96
0.98
3.26
13 12 11 10 9 8 7 6 5 4 3 2 1 0 -1
f1 (ppm)

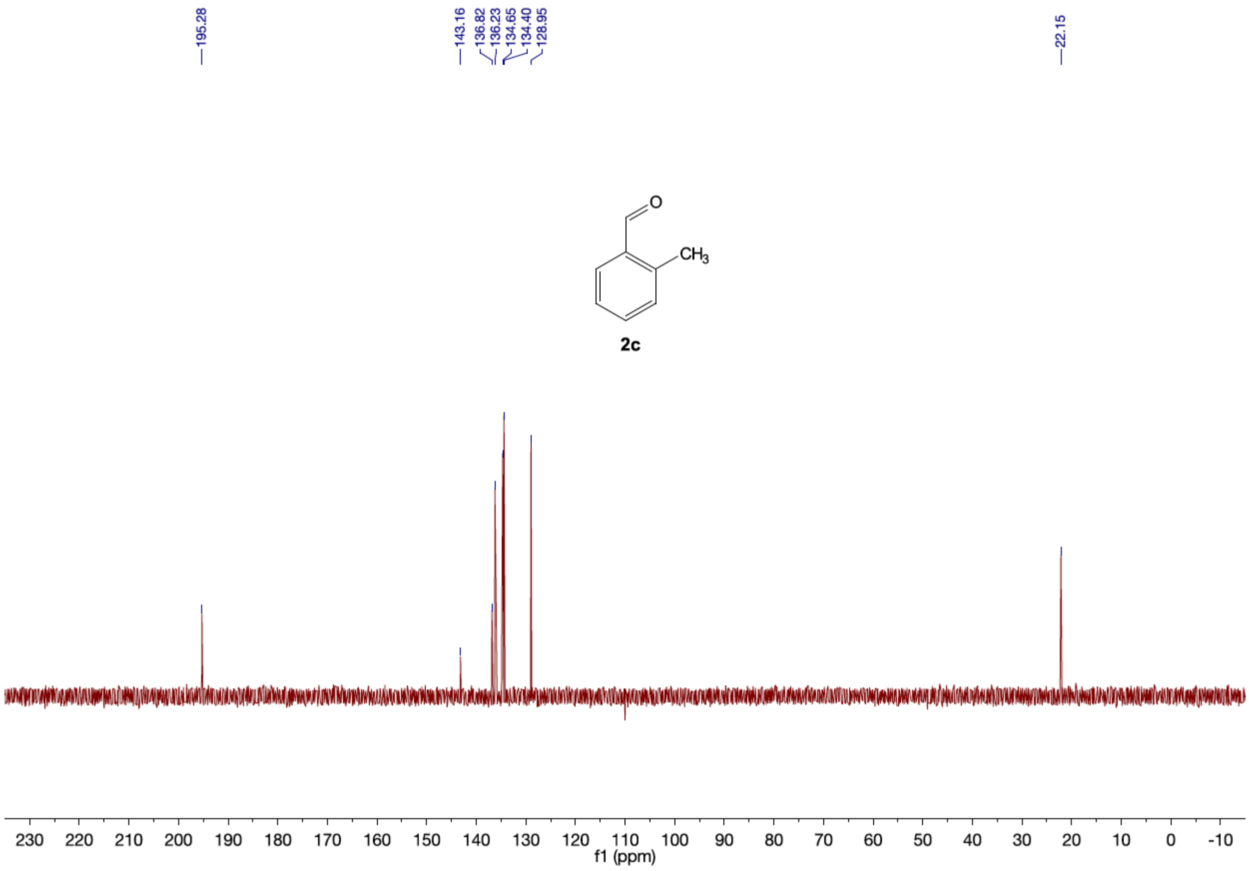
195.28
143.16
136.82
136.23
134.65
134.40
128.95
22.15
O
CH3
2c
230 220 210 200 190 180 170 160 150 140 130 120 110 100 90 80 70 60 50 40 30 20 10 0 -10
f1 (ppm)

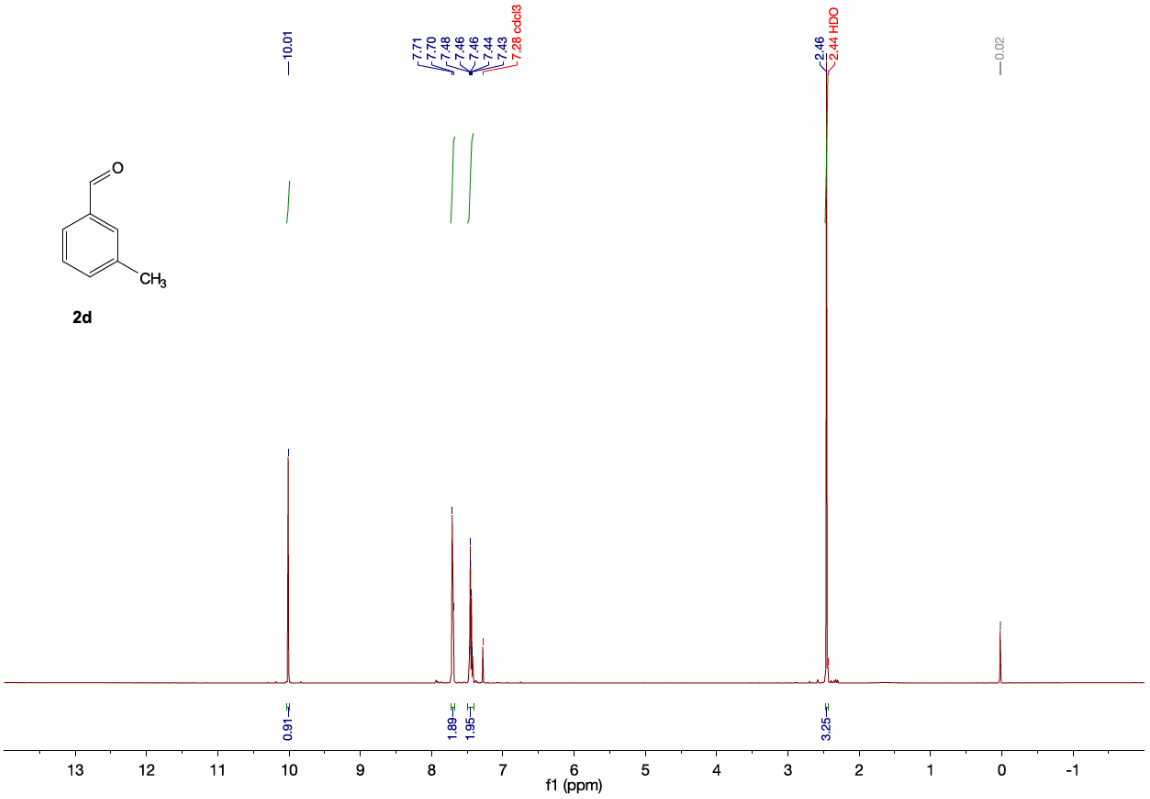

10.01
7.71
7.70
7.48
7.46
7.46
7.44
7.43
7.28 cdcl3
2.46
2.44 HDO
0.02
O
CH3
2d
0.91
1.89
1.95
3.25
13
12
11
10
9
8
7
6
5
4
3
2
1
0
-1
f1 (ppm)

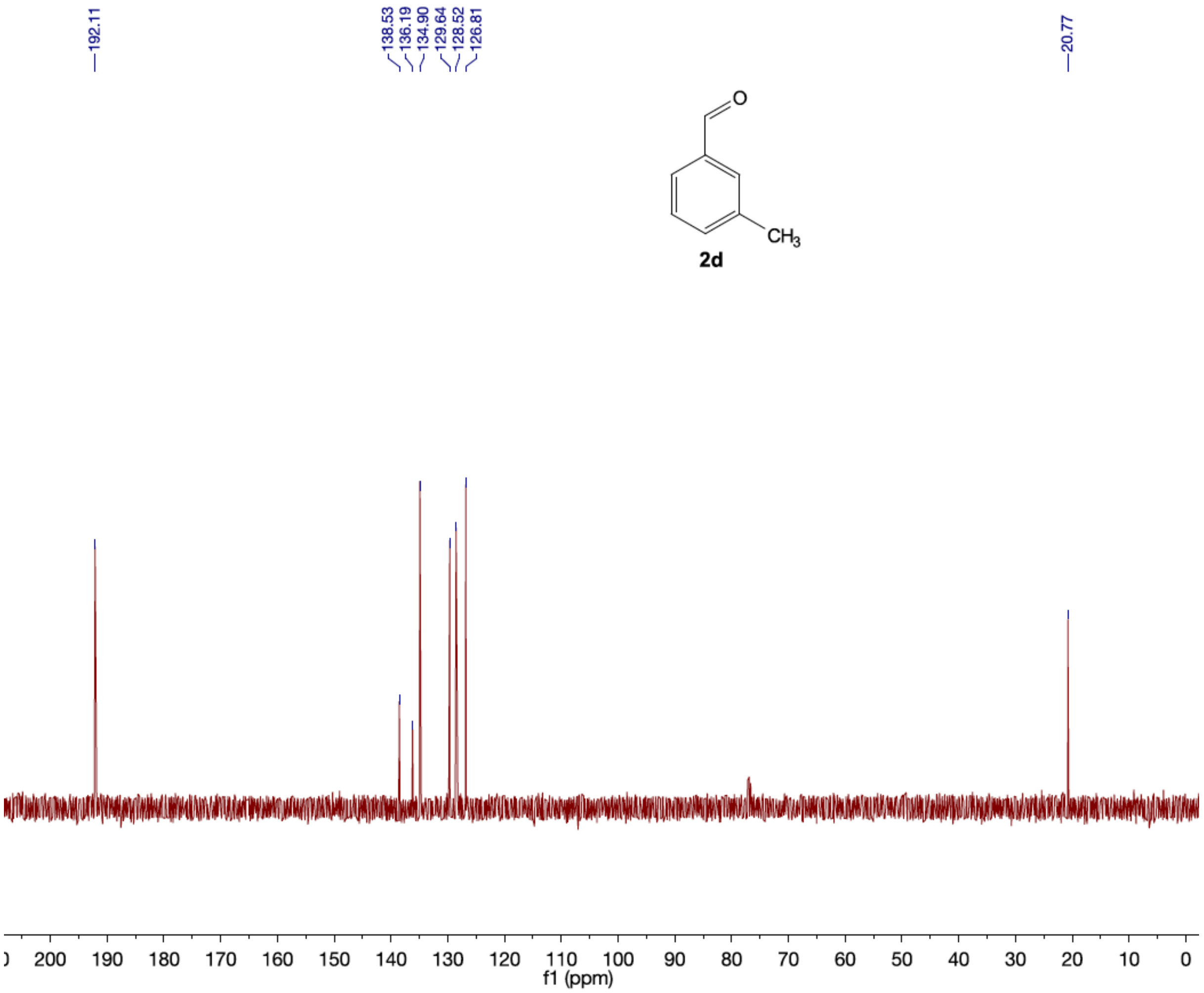

192.11
138.53
136.19
134.90
129.64
128.52
126.81
20.77
O
CH3
2d
200
190
180
170
160
150
140
130
120
110
100
90
80
70
60
50
40
30
20
10
0
f1 (ppm)

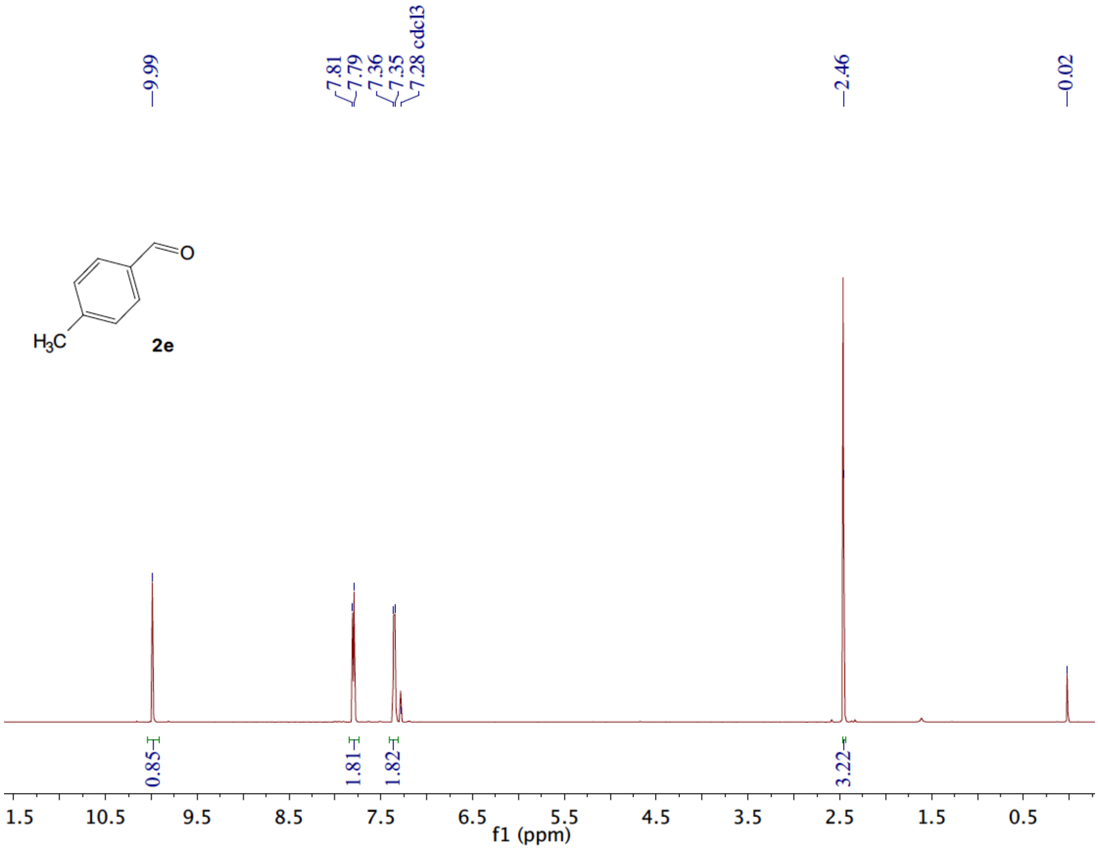

9.99
7.81
7.79
7.36
7.35
7.28 cdcl3
2.46
0.02
H3C
O
2e
0.85
1.81
1.82
3.22
1.5 10.5 9.5 8.5 7.5 6.5 5.5 4.5 3.5 2.5 1.5 0.5
f1 (ppm)

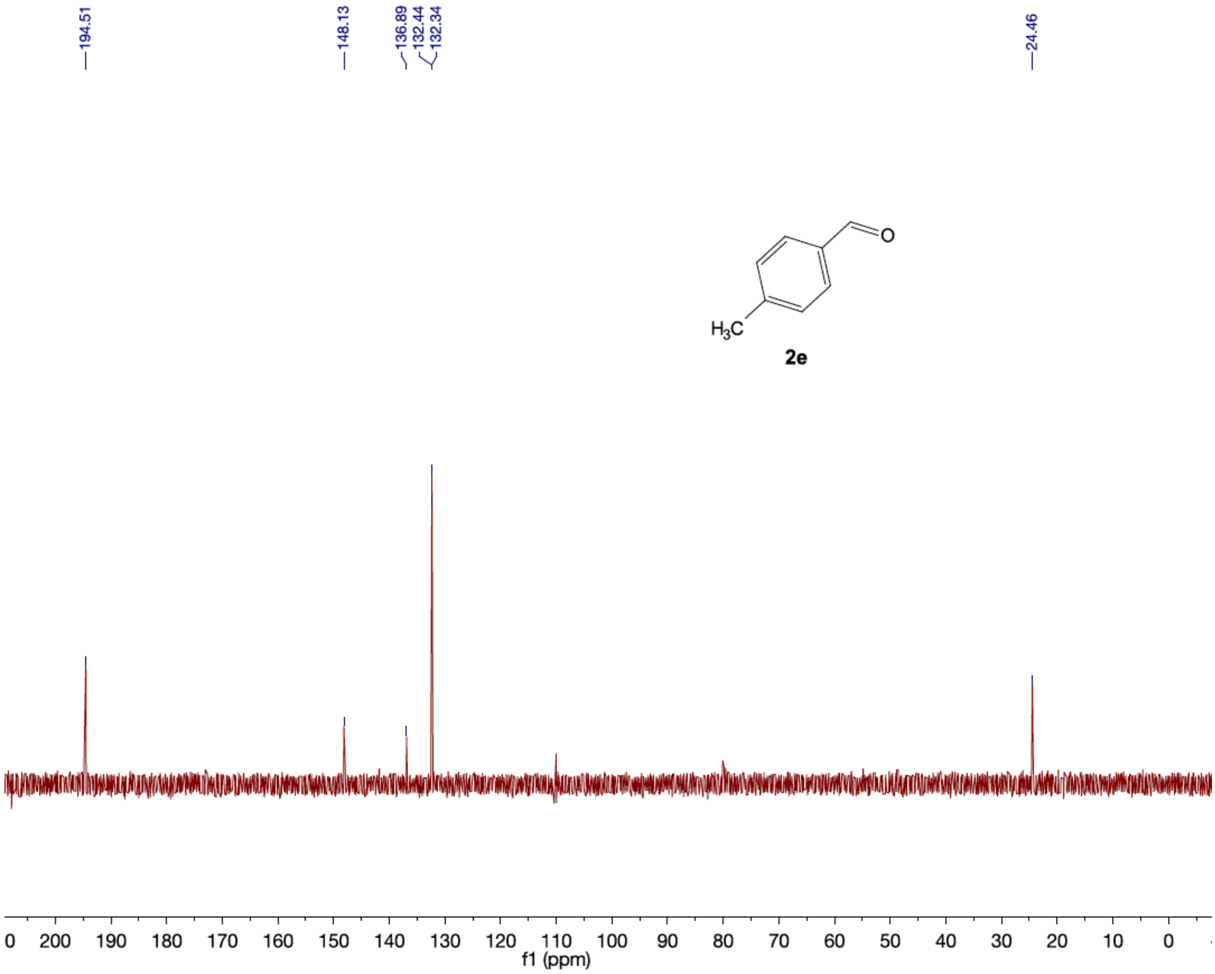

194.51
148.13
136.89
132.44
132.34
24.46
H3C
O
2e
0 200 190 180 170 160 150 140 130 120 110 100 90 80 70 60 50 40 30 20 10 0
f1 (ppm)

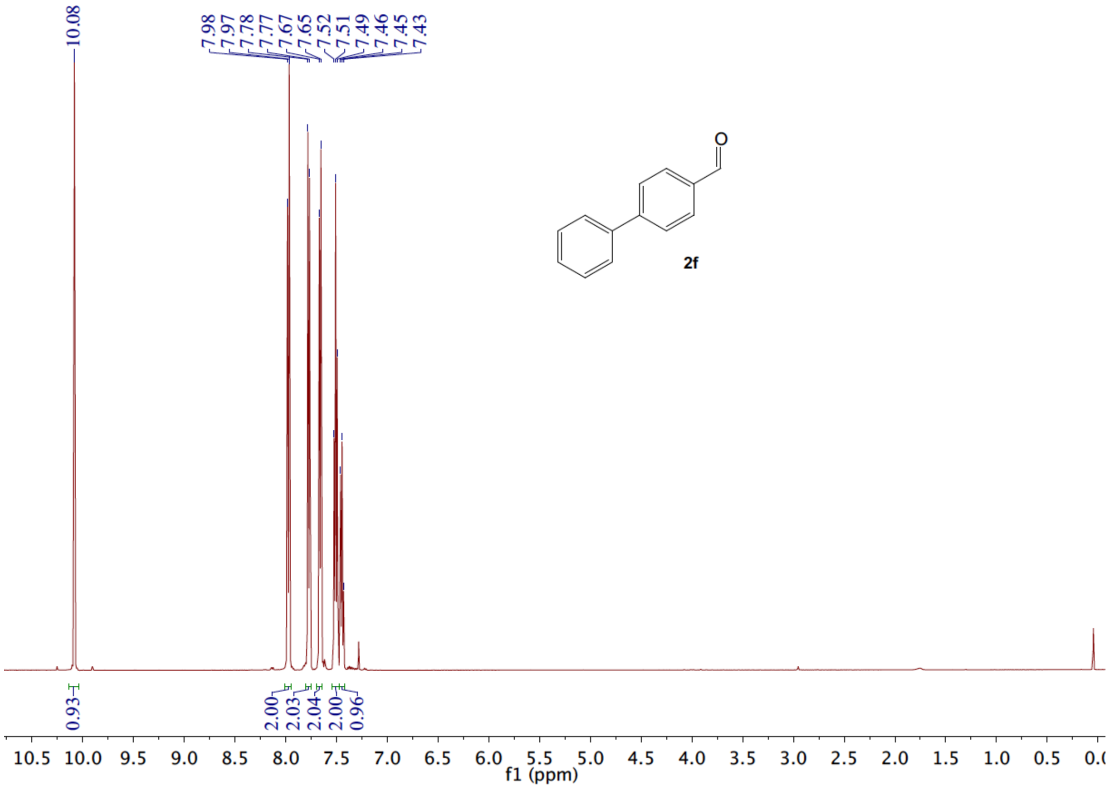

10.08
7.98 7.97 7.78 7.77 7.67 7.65 7.52 7.51 7.49 7.46 7.45 7.43
2f
0.93 2.00 2.03 2.04 2.00 0.96
10.5 10.0 9.5 9.0 8.5 8.0 7.5 7.0 6.5 6.0 5.5 5.0 4.5 4.0 3.5 3.0 2.5 2.0 1.5 1.0 0.5 0.0
f1 (ppm)

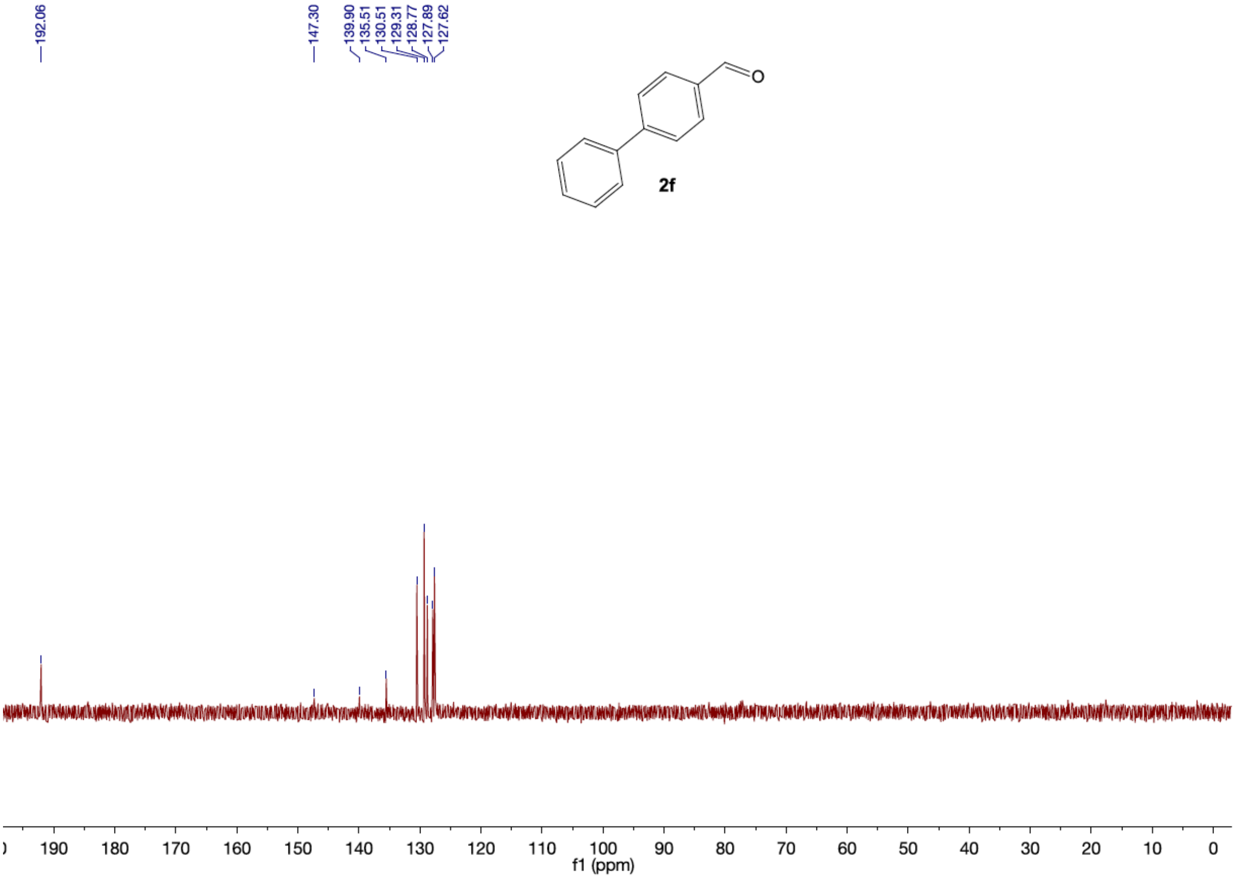

192.06
147.30
139.90 135.51 130.51 129.31 128.77 127.89 127.62
2f
190 180 170 160 150 140 130 120 110 100 90 80 70 60 50 40 30 20 10 0
f1 (ppm)

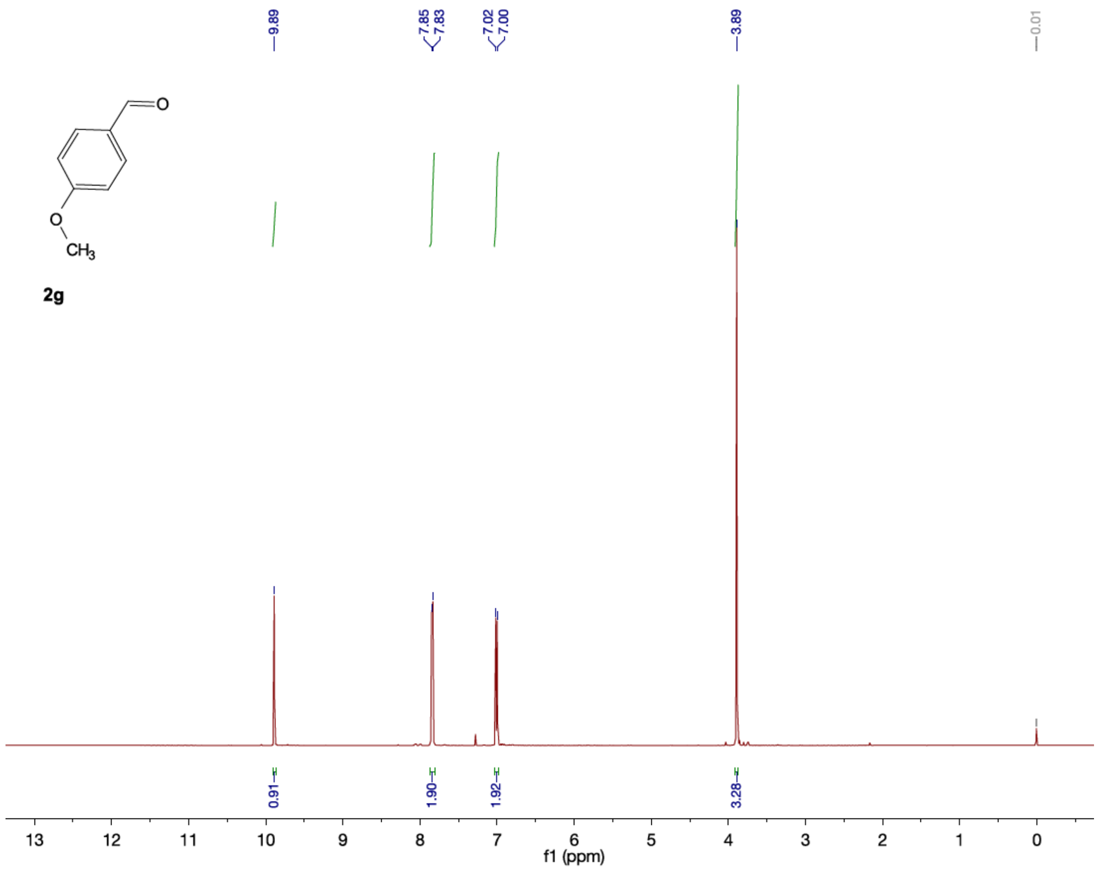
9.89
7.85
7.83
7.02
7.00
3.89
0.01
O
O
CH3
0.91
1.90
1.92
3.28
13
12
11
10
9
8
7
6
5
4
3
2
1
0
f1 (ppm)

2g

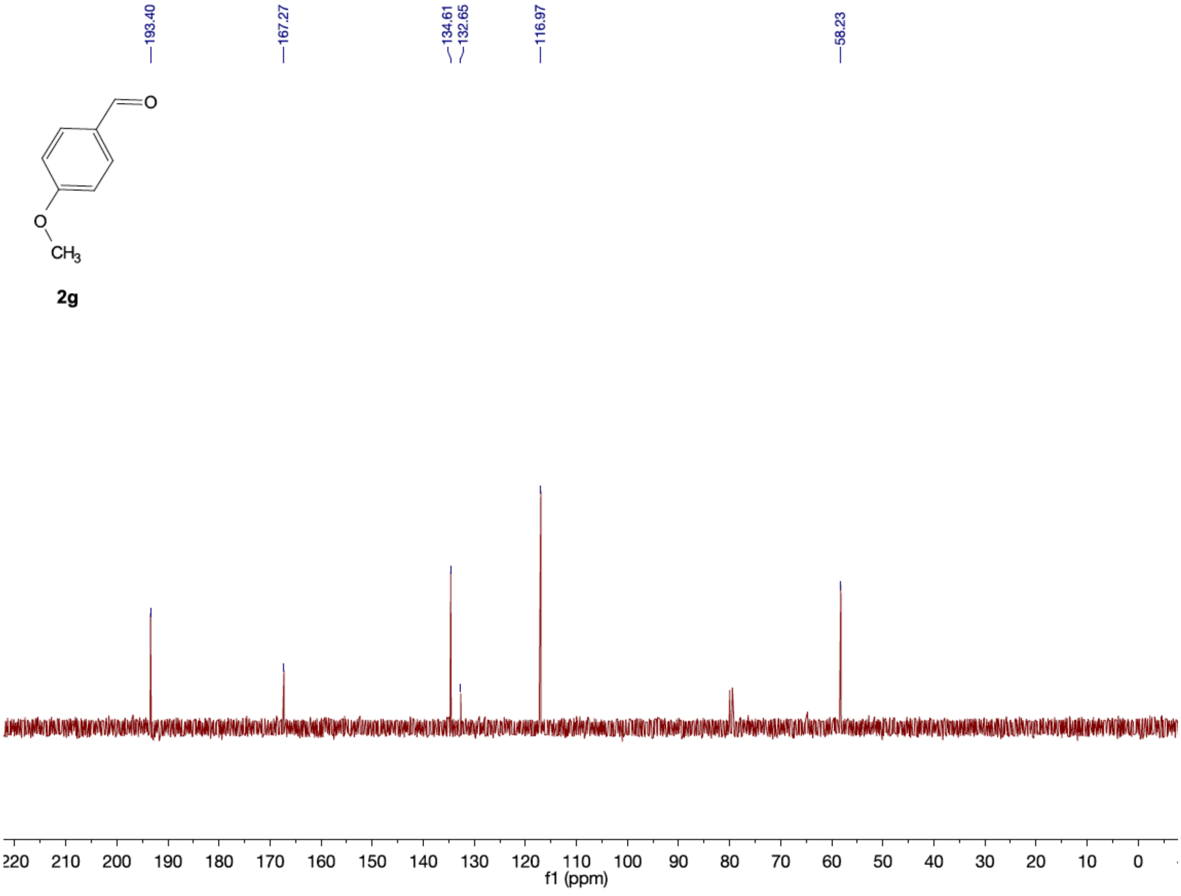
193.40
167.27
134.61
132.65
116.97
58.23
O
O
CH3
220
210
200
190
180
170
160
150
140
130
120
110
100
90
80
70
60
50
40
30
20
10
0
f1 (ppm)

2g

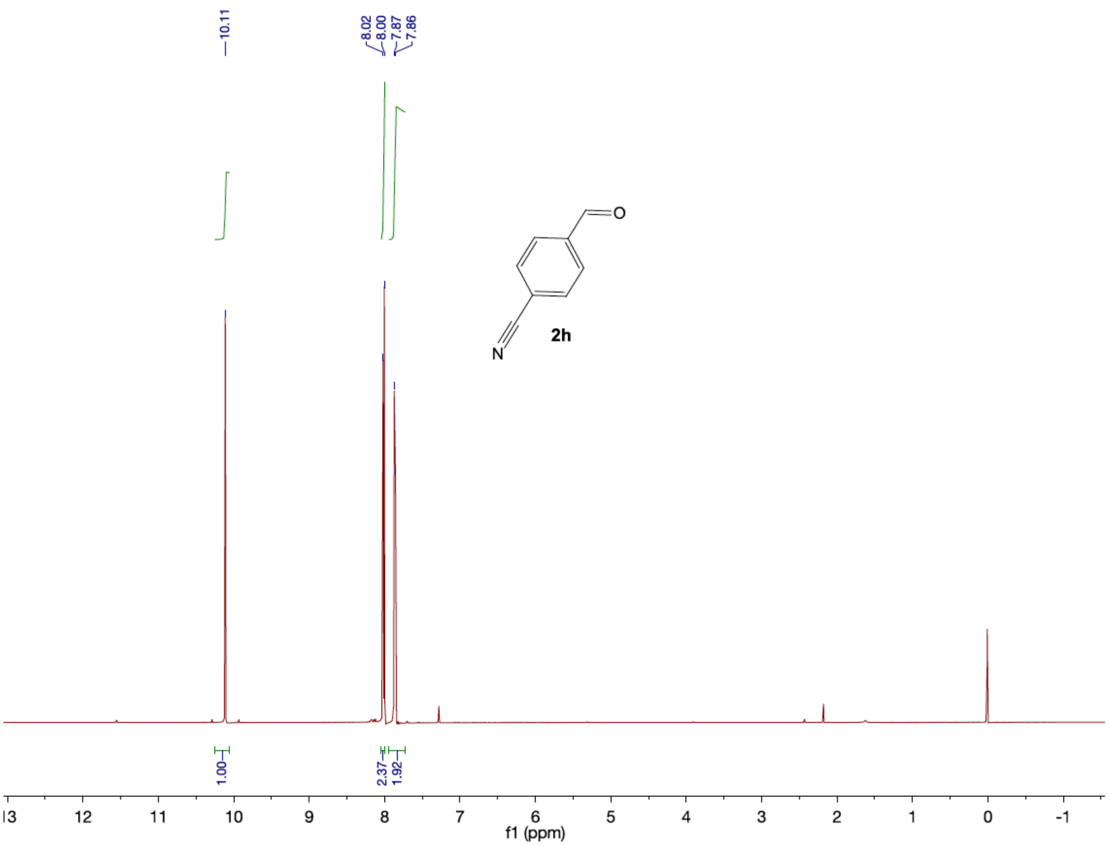
10.11
8.02
8.00
7.87
7.86
2h
1.00
2.37
1.92
13
12
11
10
9
8
7
6
5
4
3
2
1
0
-1
f1 (ppm)

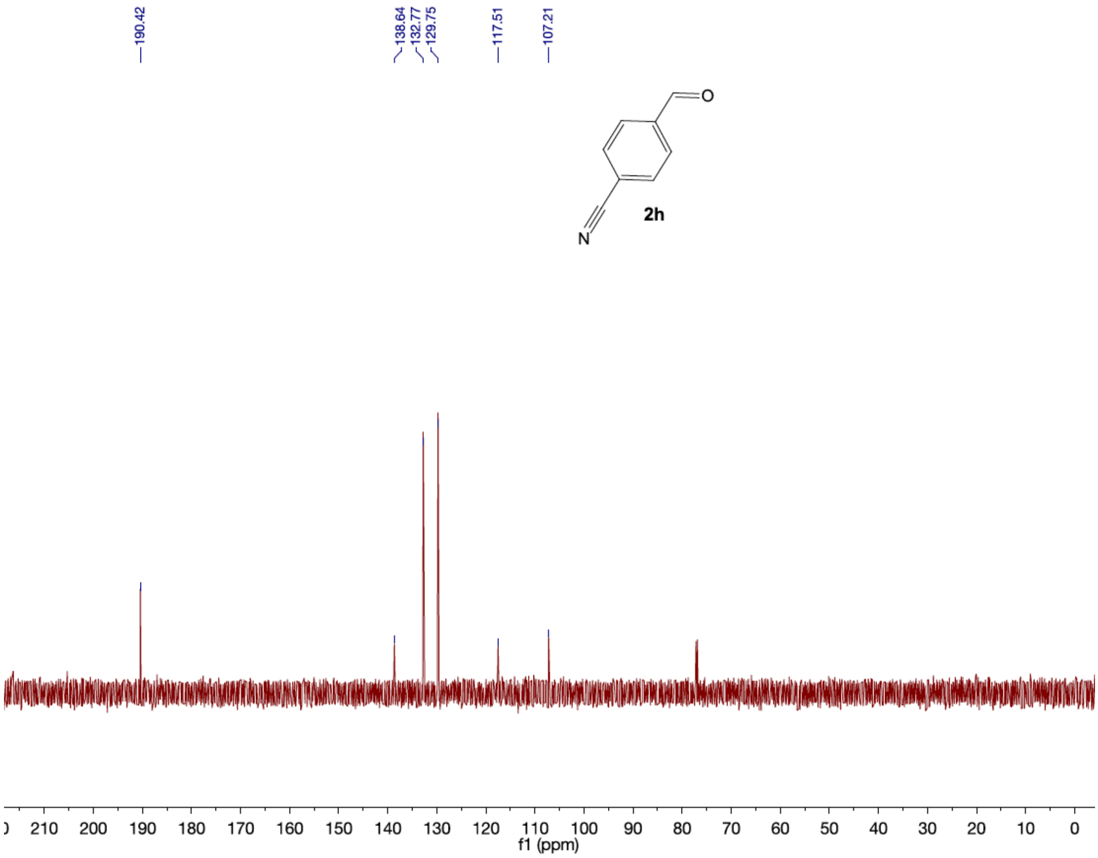
190.42
138.64
132.77
129.75
117.51
107.21
2h
210
200
190
180
170
160
150
140
130
120
110
100
90
80
70
60
50
40
30
20
10
0
f1 (ppm)

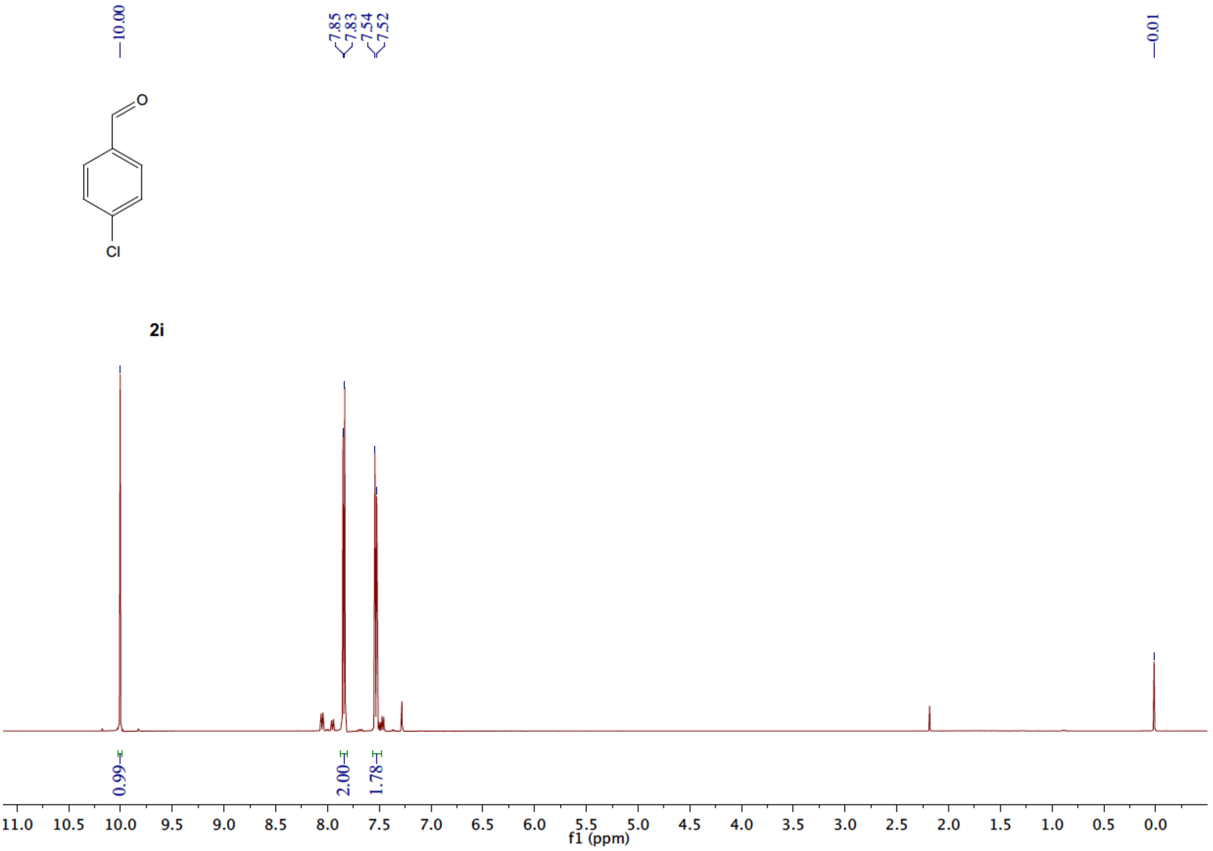
10.00
7.85
7.83
7.54
7.52
0.01
O
Cl
0.99
2.00
1.78
11.0 10.5 10.0 9.5 9.0 8.5 8.0 7.5 7.0 6.5 6.0 5.5 5.0 4.5 4.0 3.5 3.0 2.5 2.0 1.5 1.0 0.5 0.0
f1 (ppm)

2i

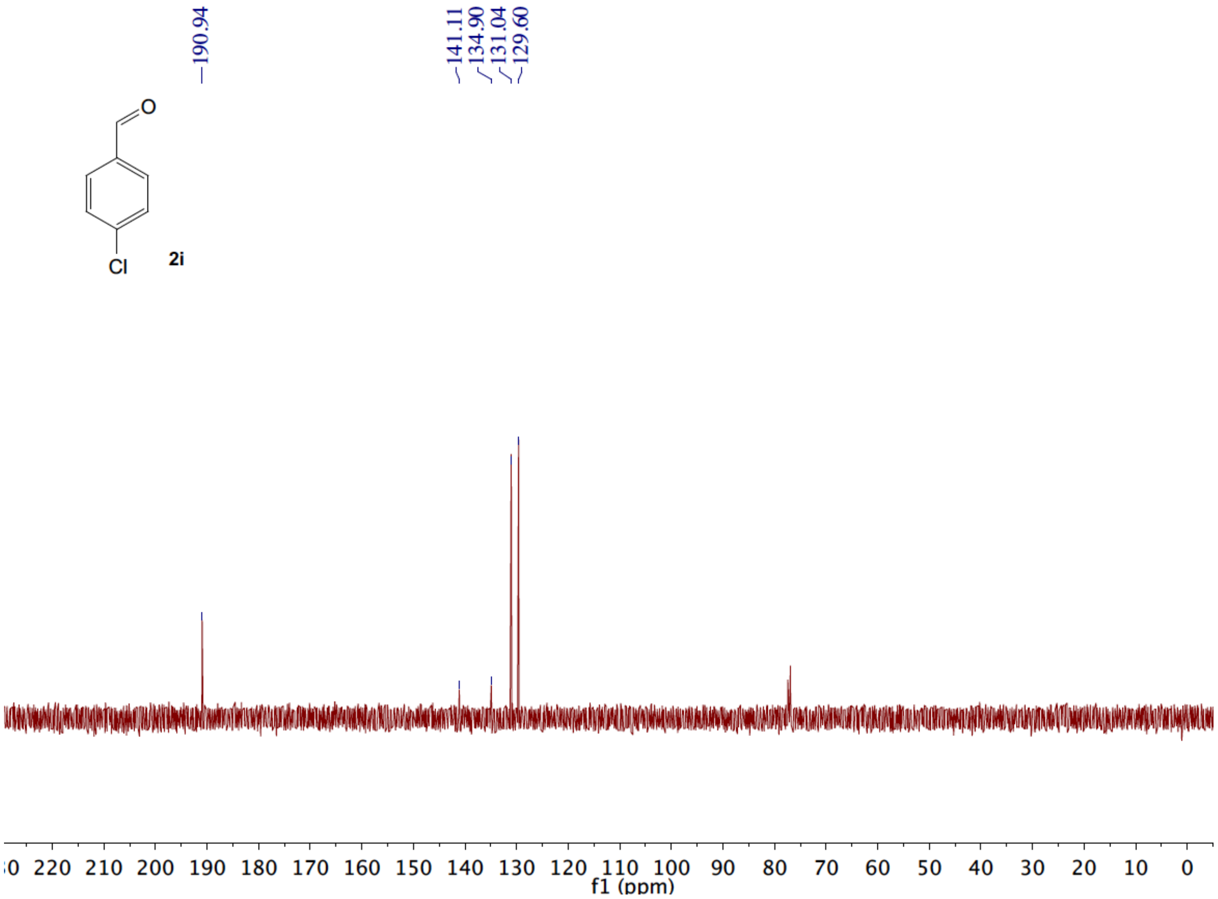
190.94
141.11
134.90
131.04
129.60
O
Cl
2i
220 210 200 190 180 170 160 150 140 130 120 110 100 90 80 70 60 50 40 30 20 10 0
f1 (ppm)

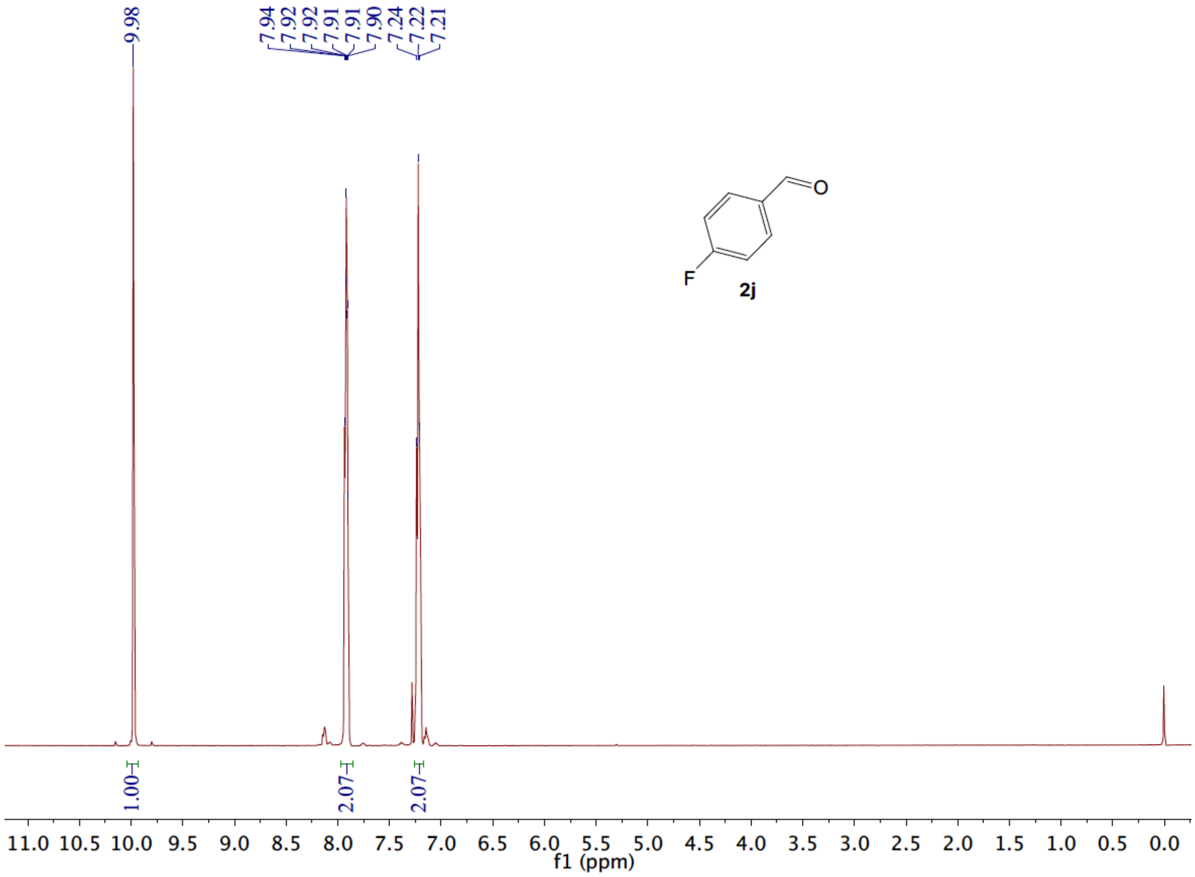
9.98
7.94
7.92
7.92
7.91
7.91
7.90
7.24
7.22
7.21
O
F
2j
1.00
2.07
2.07
11.0 10.5 10.0 9.5 9.0 8.5 8.0 7.5 7.0 6.5 6.0 5.5 5.0 4.5 4.0 3.5 3.0 2.5 2.0 1.5 1.0 0.5 0.0
f1 (ppm)

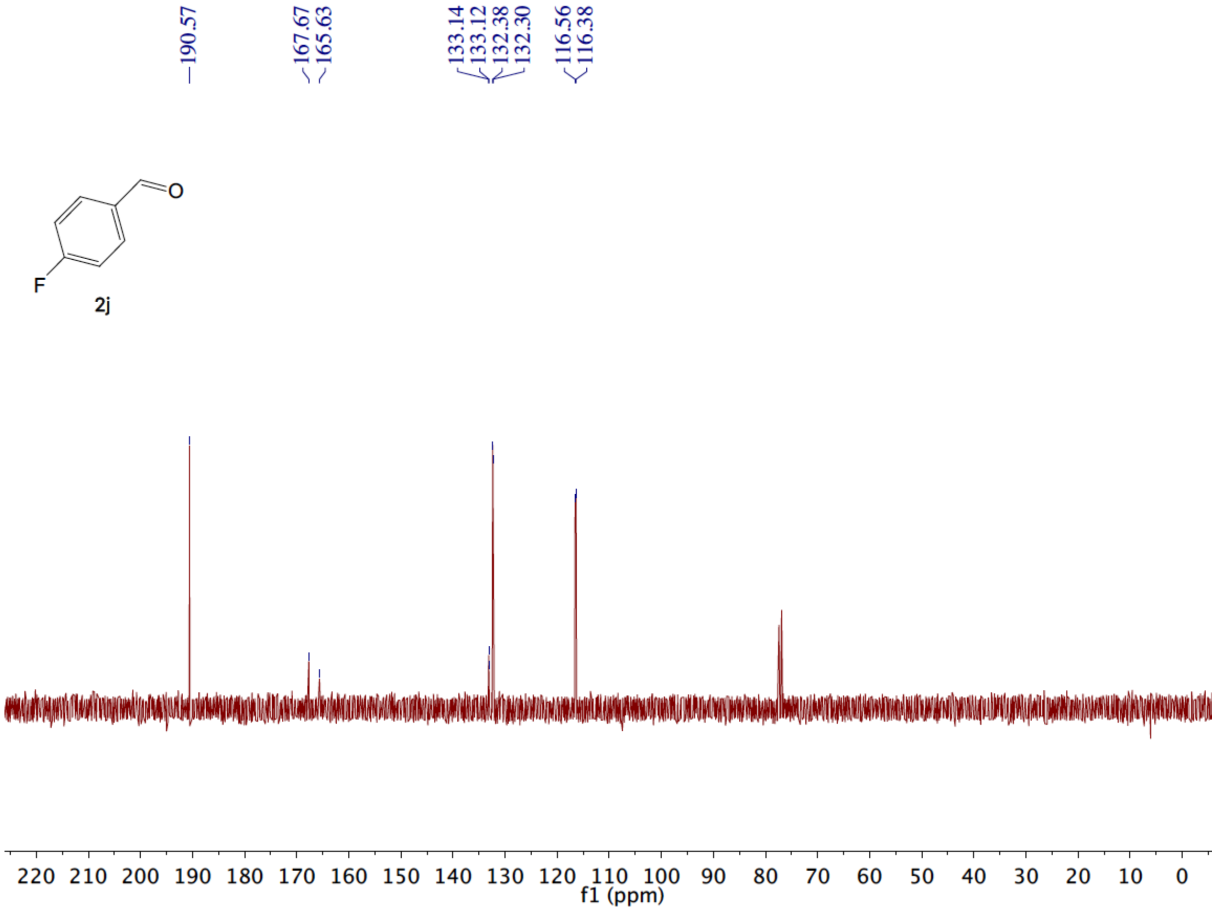
190.57
167.67
165.63
133.14
133.12
132.38
132.30
116.56
116.38
O
F
2j
220 210 200 190 180 170 160 150 140 130 120 110 100 90 80 70 60 50 40 30 20 10 0
f1 (ppm)

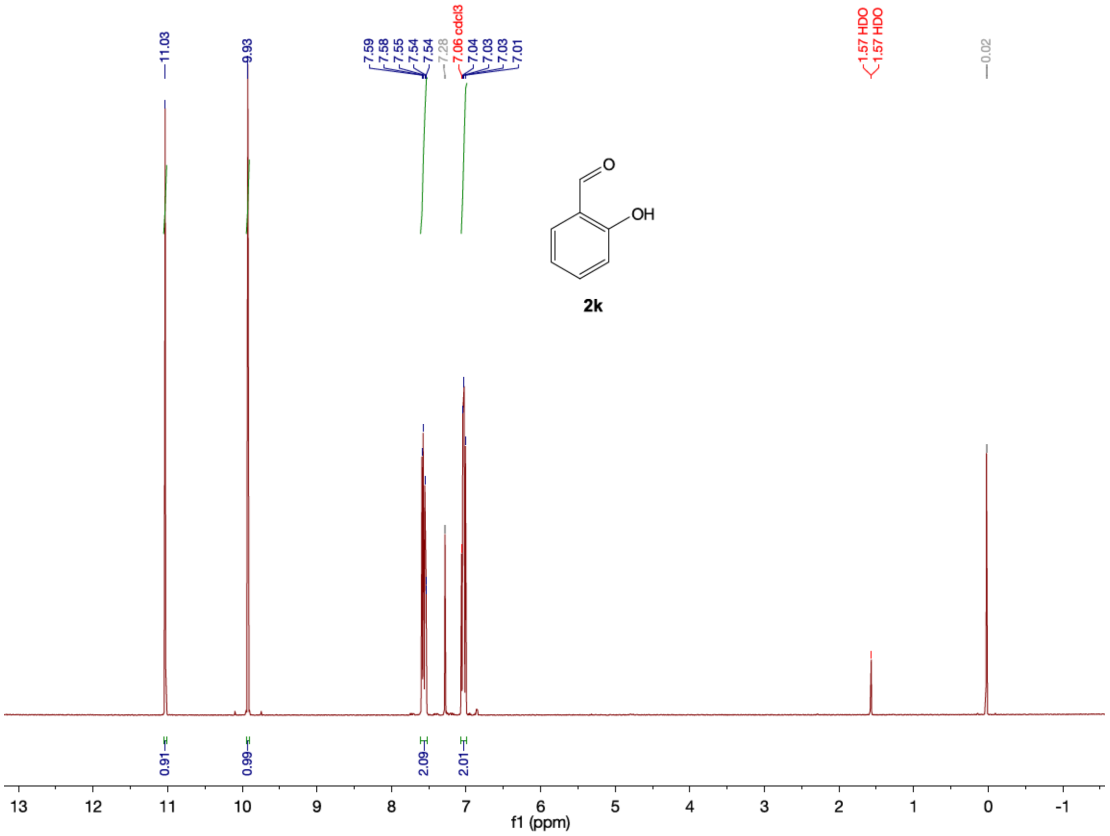
11.03
9.93
7.59
7.58
7.55
7.54
7.54
7.28
7.06 cdcl3
7.04
7.03
7.03
7.01
1.57 HDO
1.57 HDO
0.02
O
OH
0.91
0.99
2.09
2.01
f1 (ppm)

2k

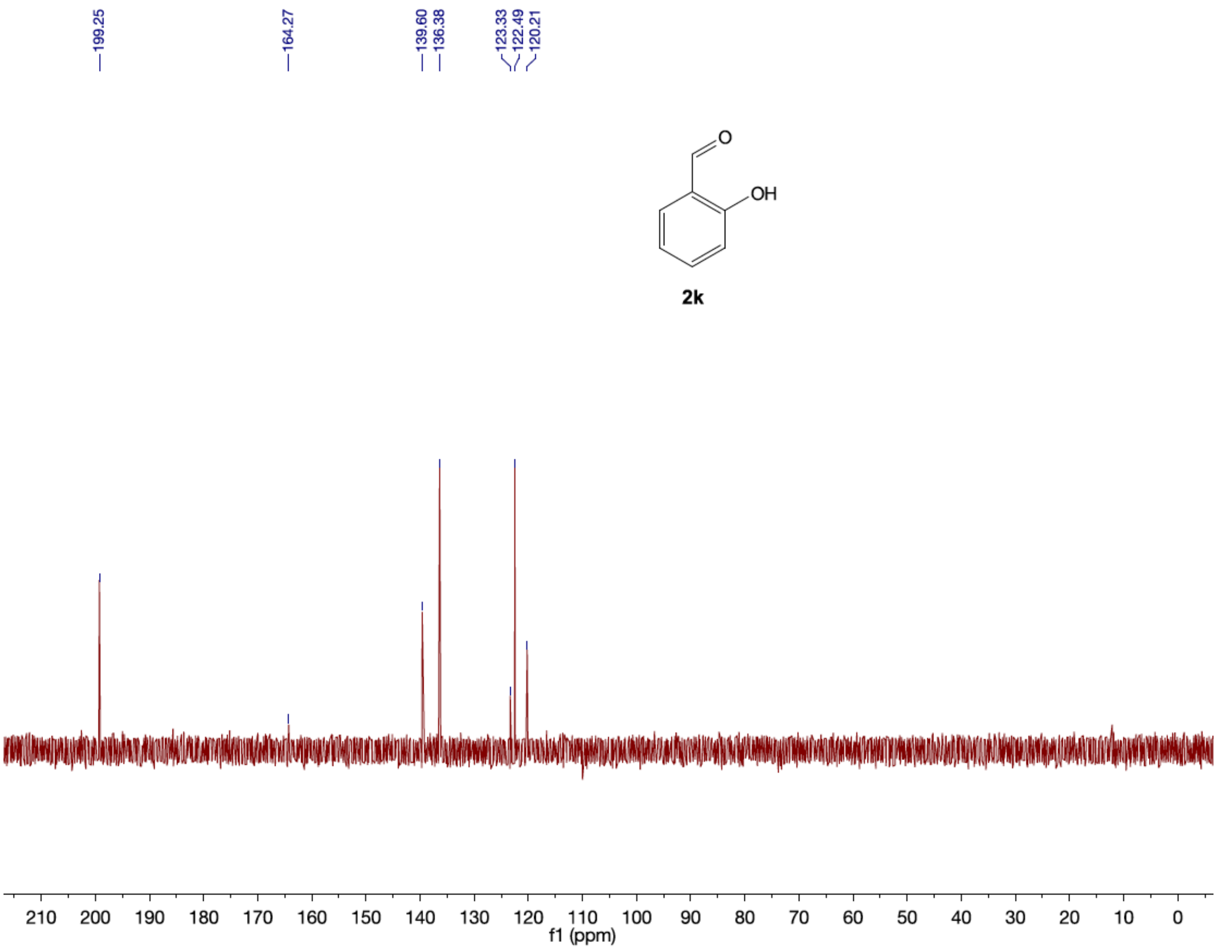
199.25
164.27
139.60
136.38
123.33
122.49
120.21
O
OH
f1 (ppm)

2k

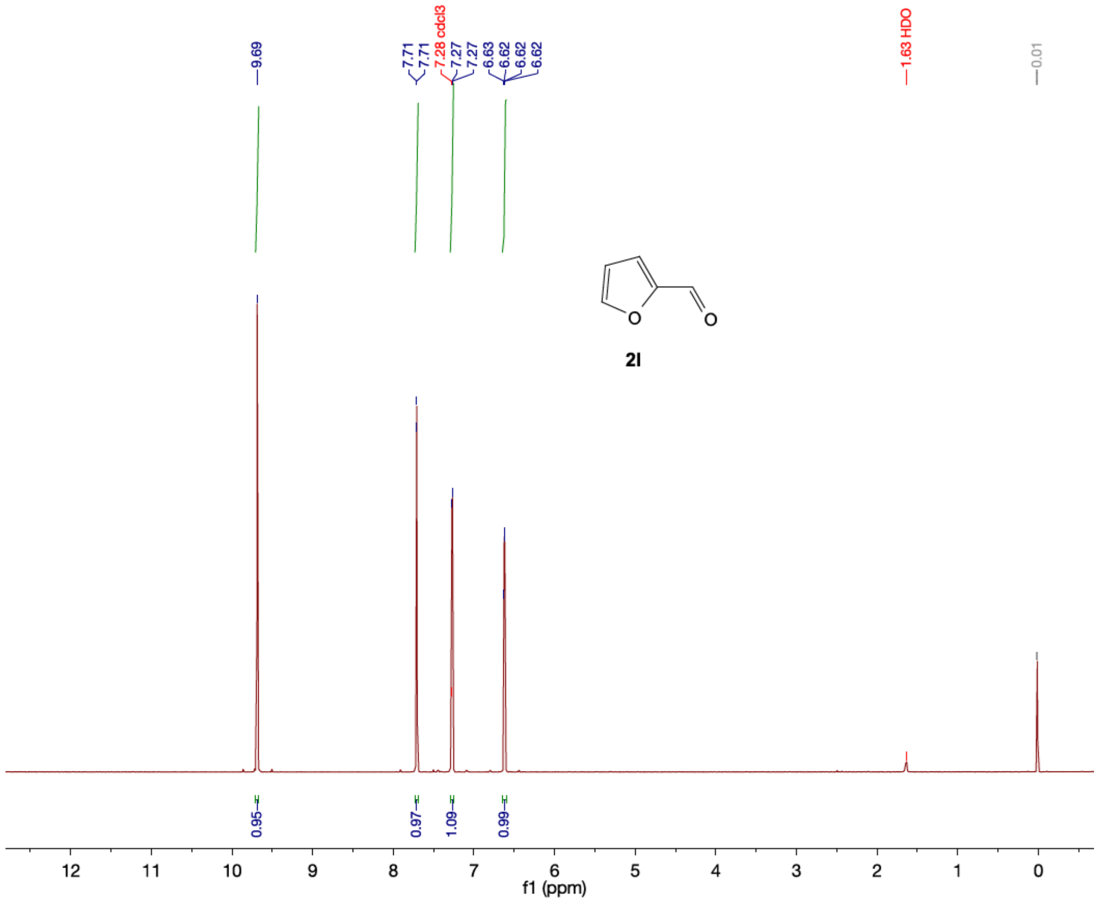
9.69
7.71
7.71
7.28 cdcl3
7.27
7.27
6.63
6.62
6.62
6.62
1.63 HDO
-0.01
O
O
2l
0.95
0.97
1.09
0.99
12 11 10 9 8 7 6 5 4 3 2 1 0
f1 (ppm)

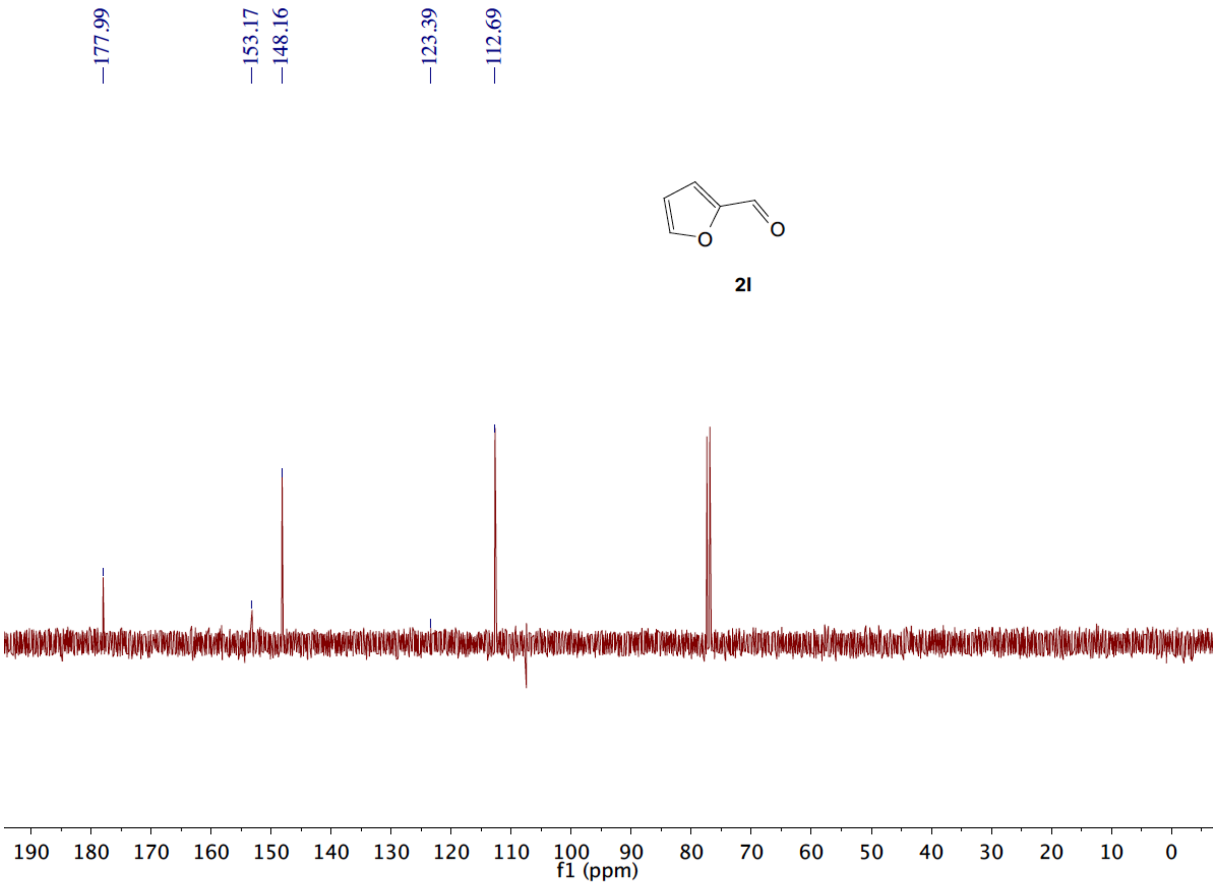
177.99
153.17
148.16
123.39
112.69
O
O
2l
190 180 170 160 150 140 130 120 110 100 90 80 70 60 50 40 30 20 10 0
f1 (ppm)

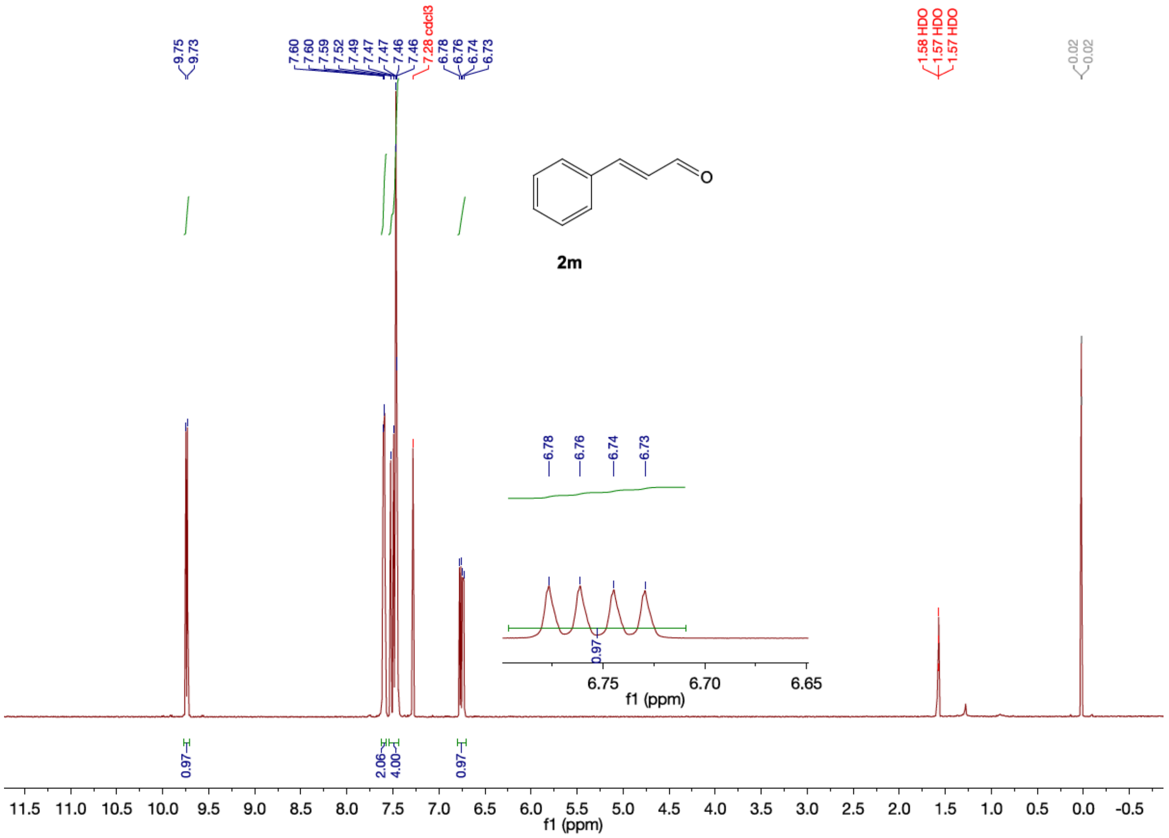
9.75
9.73
7.60
7.60
7.59
7.52
7.49
7.47
7.47
7.46
7.46
7.28 cdcl3
6.78
6.76
6.74
6.73
1.58 HDO
1.57 HDO
1.57 HDO
0.02
0.02
O
2m
0.97
2.06
4.00
0.97
6.75
6.70
6.65
f1 (ppm)
f1 (ppm)

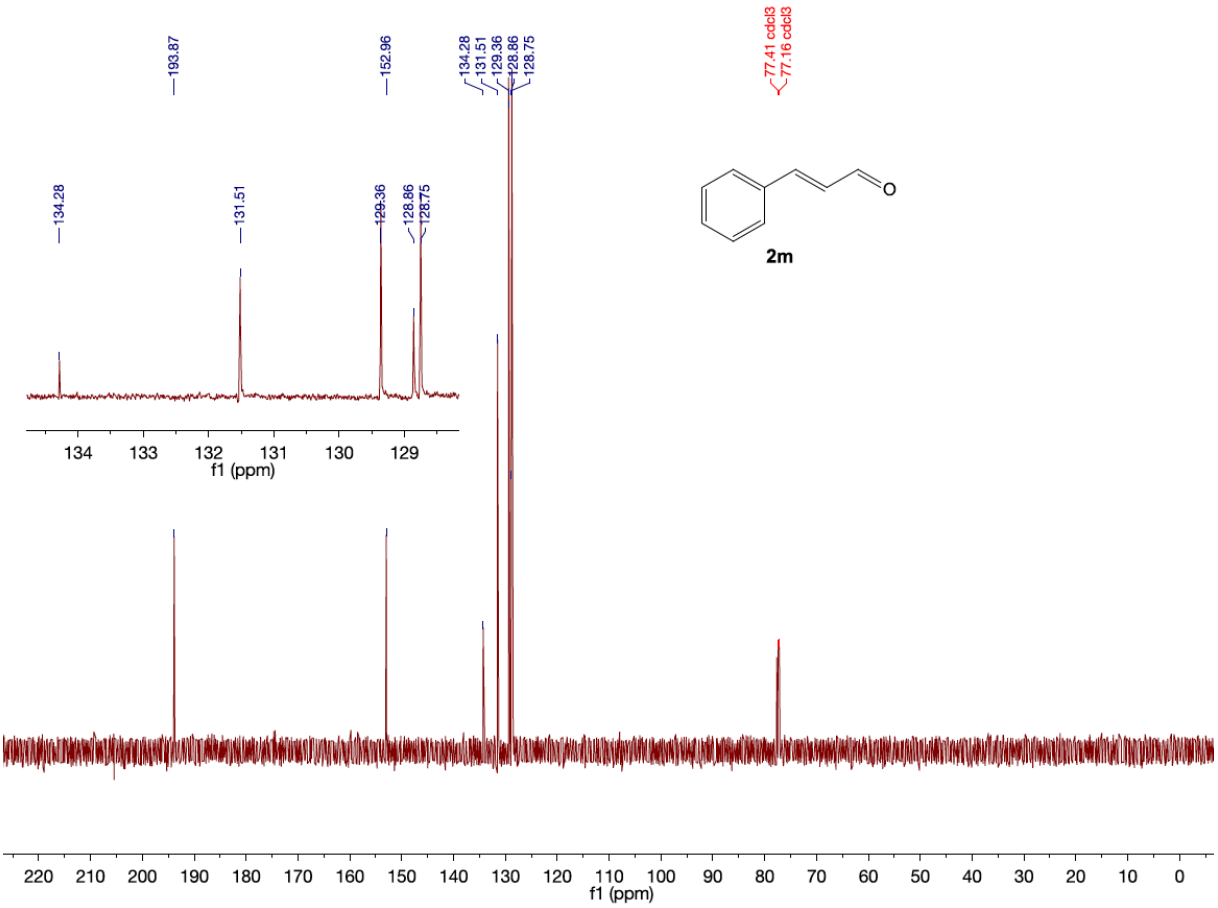
193.87
152.96
134.28
131.51
129.36
128.86
128.75
77.41 cdcl3
77.16 cdcl3
O
2m
f1 (ppm)
f1 (ppm)

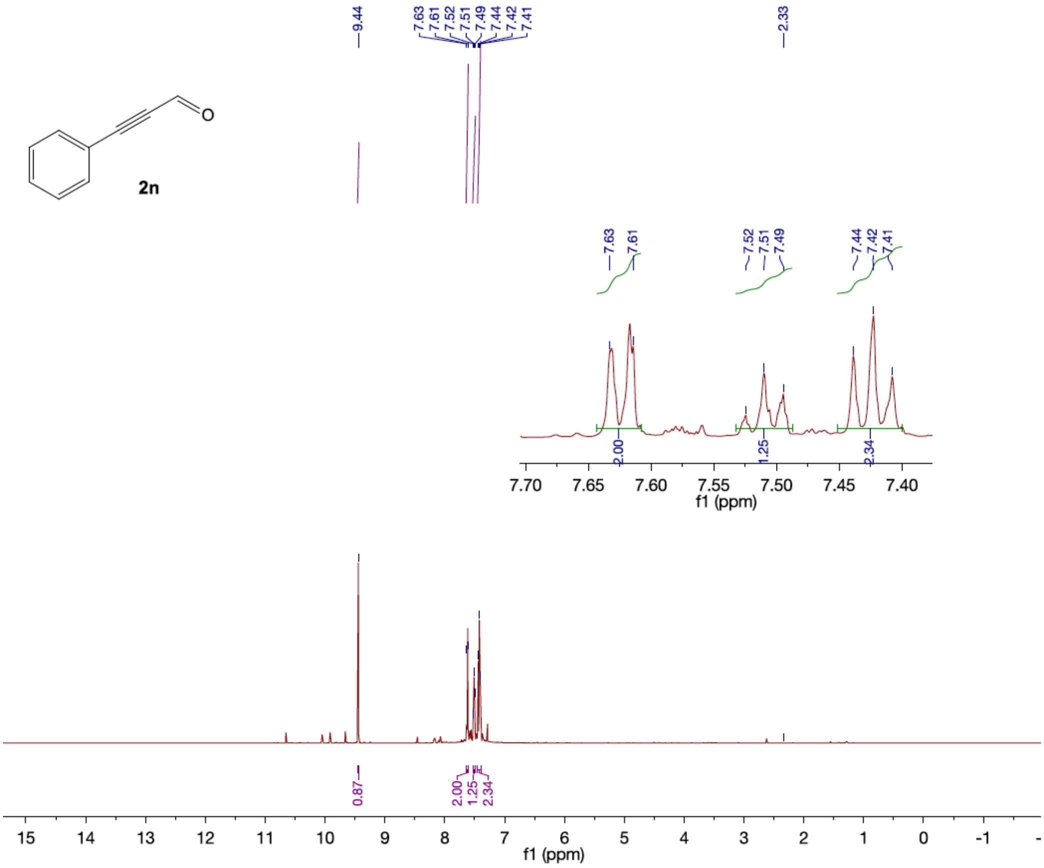
9.44
7.63
7.61
7.52
7.51
7.49
7.44
7.42
7.41
2.33
O
2n
2.00
1.25
2.34
7.70 7.65 7.60 7.55 7.50 7.45 7.40
f1 (ppm)
0.87
15 14 13 12 11 10 9 8 7 6 5 4 3 2 1 0 -1
f1 (ppm)

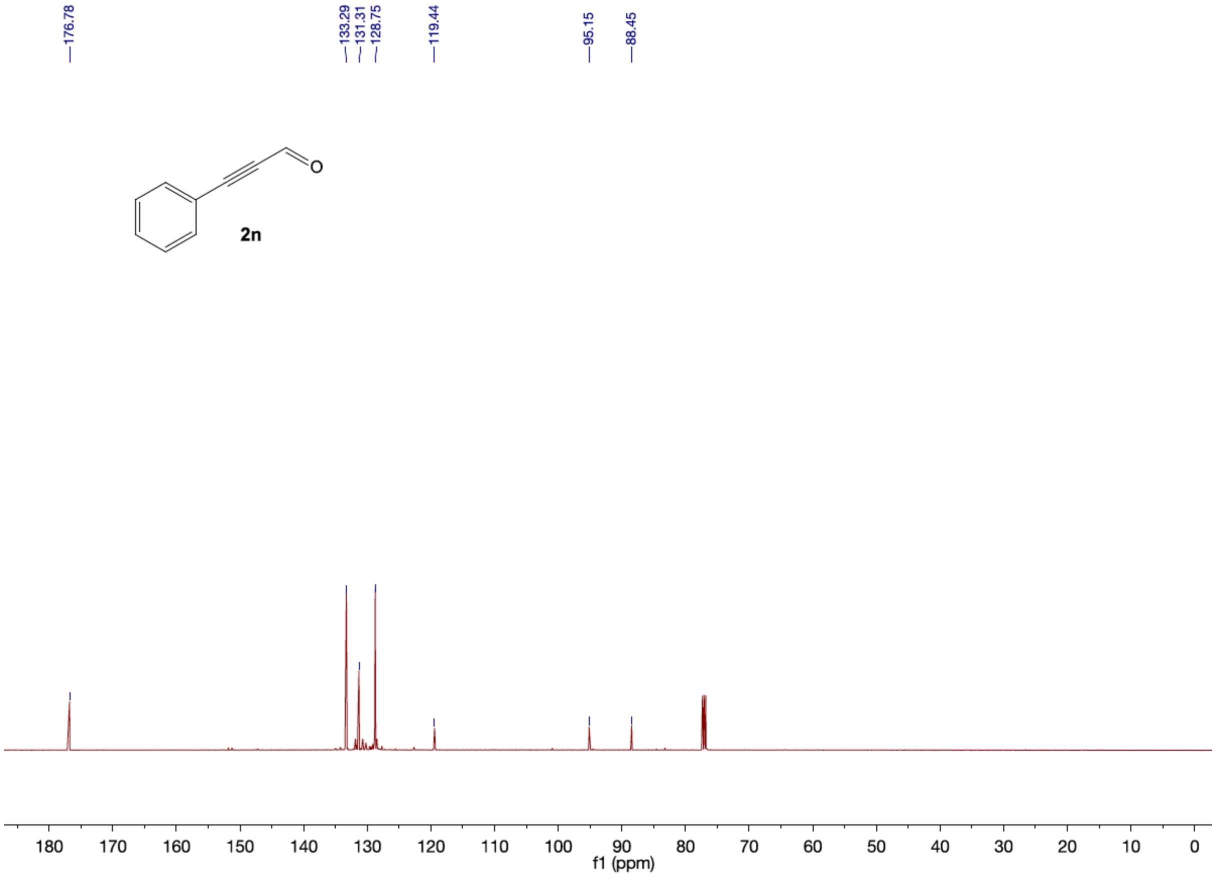
176.78
133.29
131.31
128.75
119.44
95.15
88.45
O
2n
180 170 160 150 140 130 120 110 100 90 80 70 60 50 40 30 20 10 0
f1 (ppm)

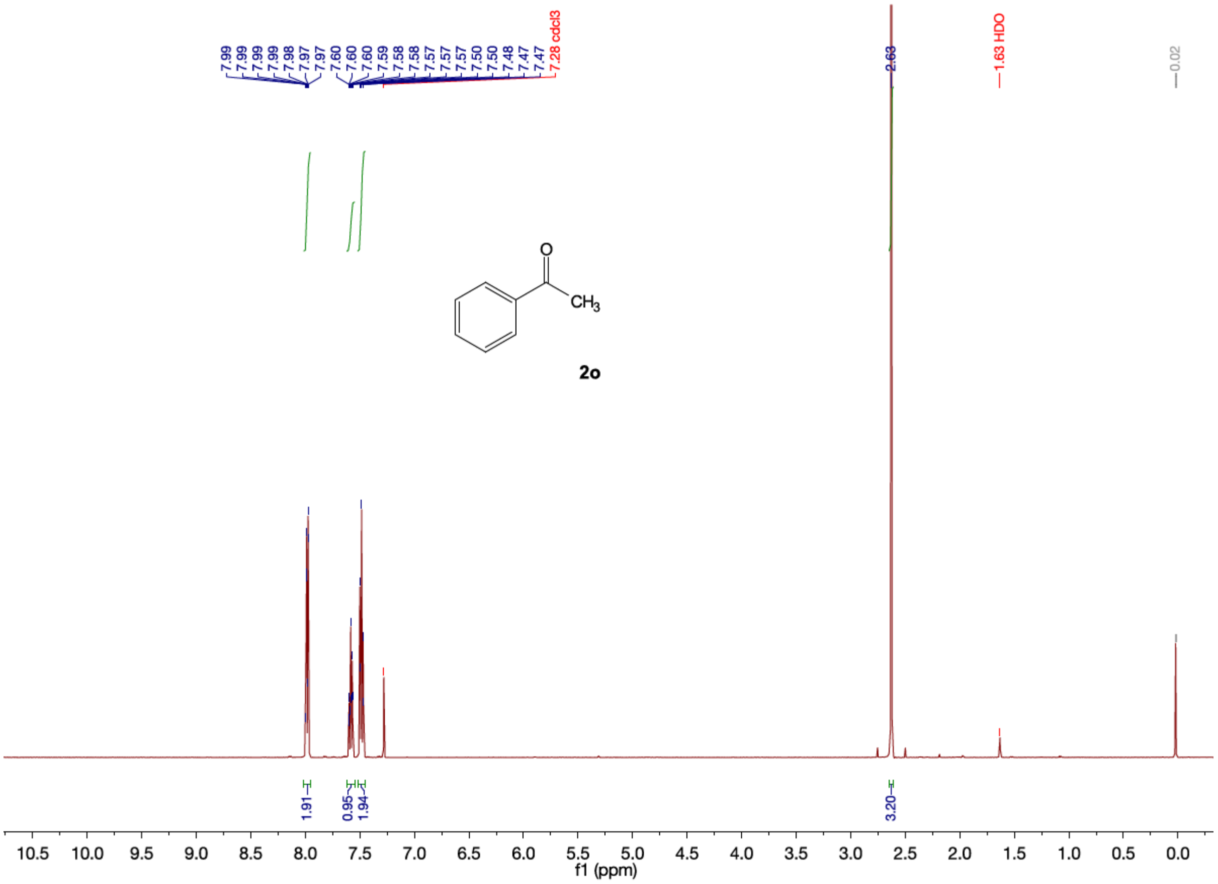
7.99
7.99
7.99
7.99
7.98
7.97
7.97
7.60
7.60
7.60
7.59
7.58
7.58
7.57
7.57
7.57
7.50
7.50
7.48
7.47
7.47
7.28 cdcl3
2.63
1.63 HDO
0.02
O
CH3
2o
1.91
0.95
1.94
3.20
10.5 10.0 9.5 9.0 8.5 8.0 7.5 7.0 6.5 6.0 5.5 5.0 4.5 4.0 3.5 3.0 2.5 2.0 1.5 1.0 0.5 0.0
f1 (ppm)

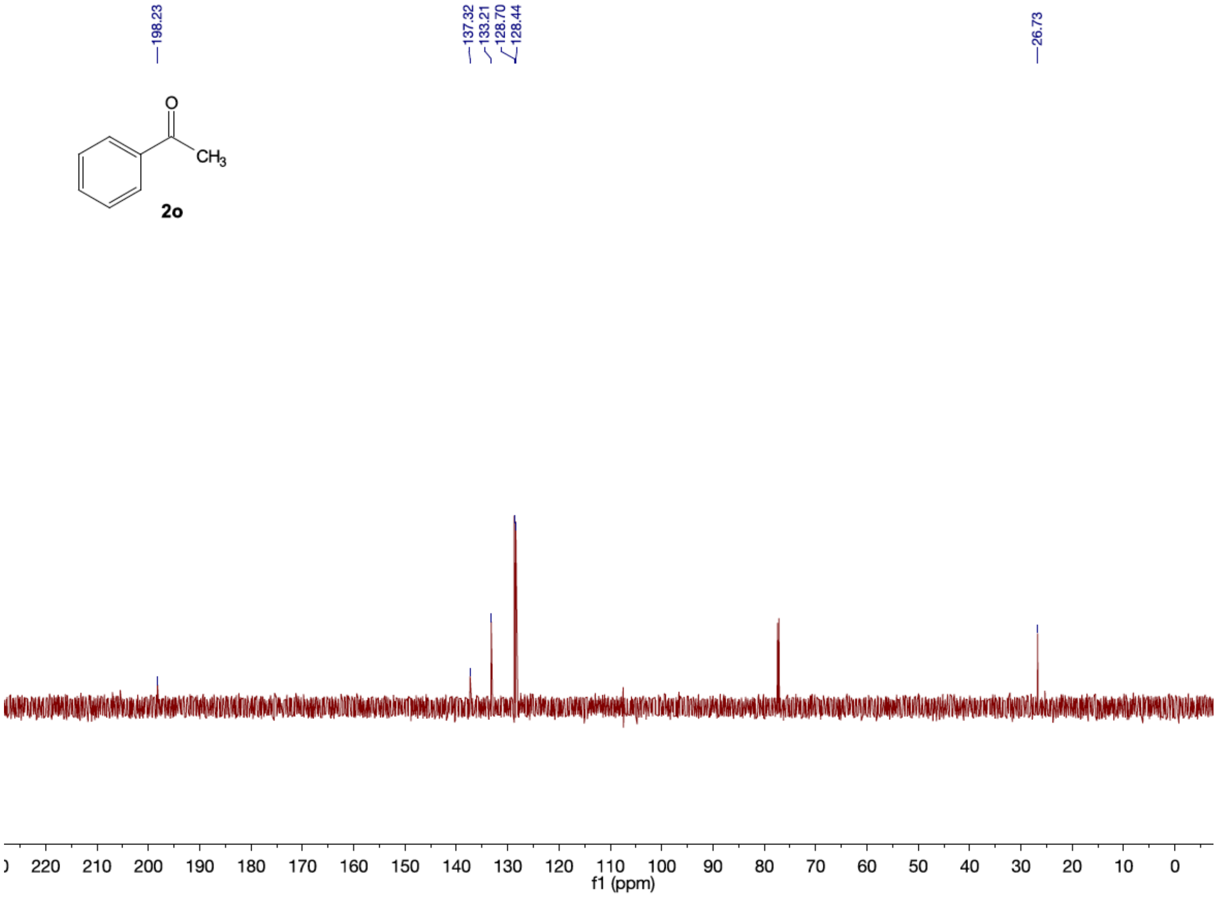
198.23
137.32
133.21
128.70
128.44
26.73
O
CH3
2o
220 210 200 190 180 170 160 150 140 130 120 110 100 90 80 70 60 50 40 30 20 10 0
f1 (ppm)

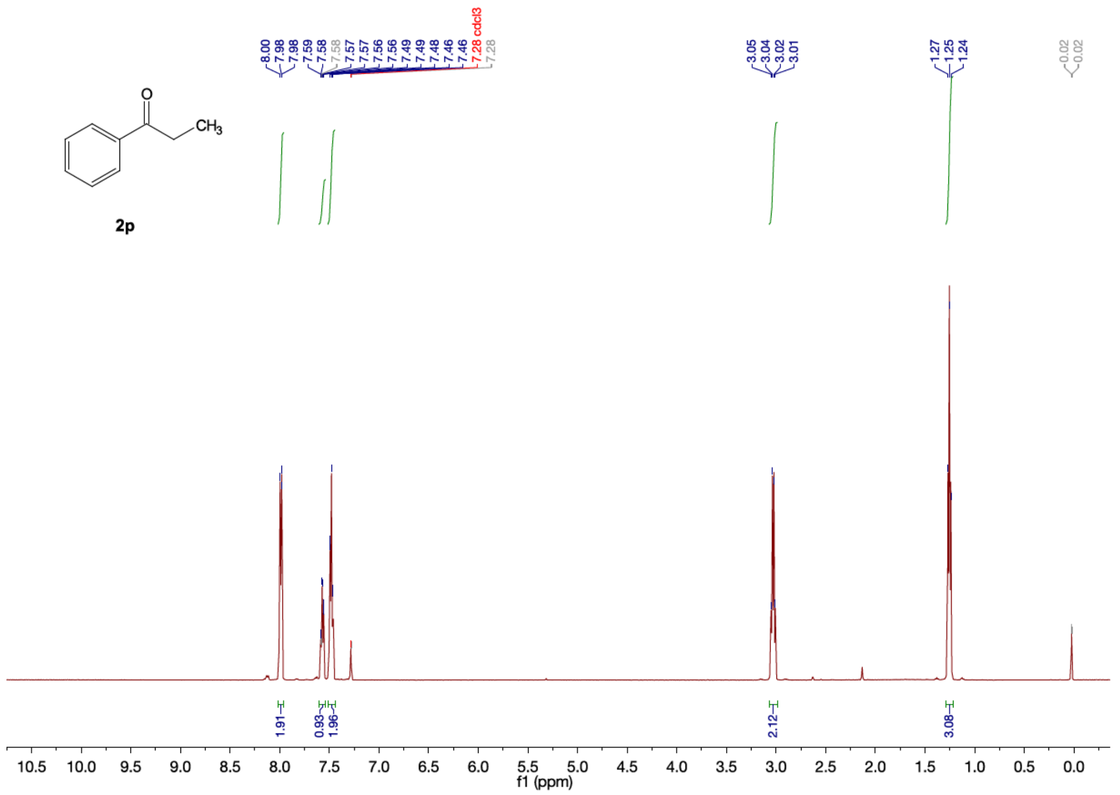
8.00
7.98
7.98
7.59
7.58
7.58
7.57
7.57
7.56
7.56
7.49
7.49
7.48
7.46
7.28 cdcl3
7.28
3.05
3.04
3.02
3.01
1.27
1.25
1.24
0.02
0.02
O
CH3
2p
1.91
0.93
1.96
2.12
3.08
10.5 10.0 9.5 9.0 8.5 8.0 7.5 7.0 6.5 6.0 5.5 5.0 4.5 4.0 3.5 3.0 2.5 2.0 1.5 1.0 0.5 0.0
f1 (ppm)

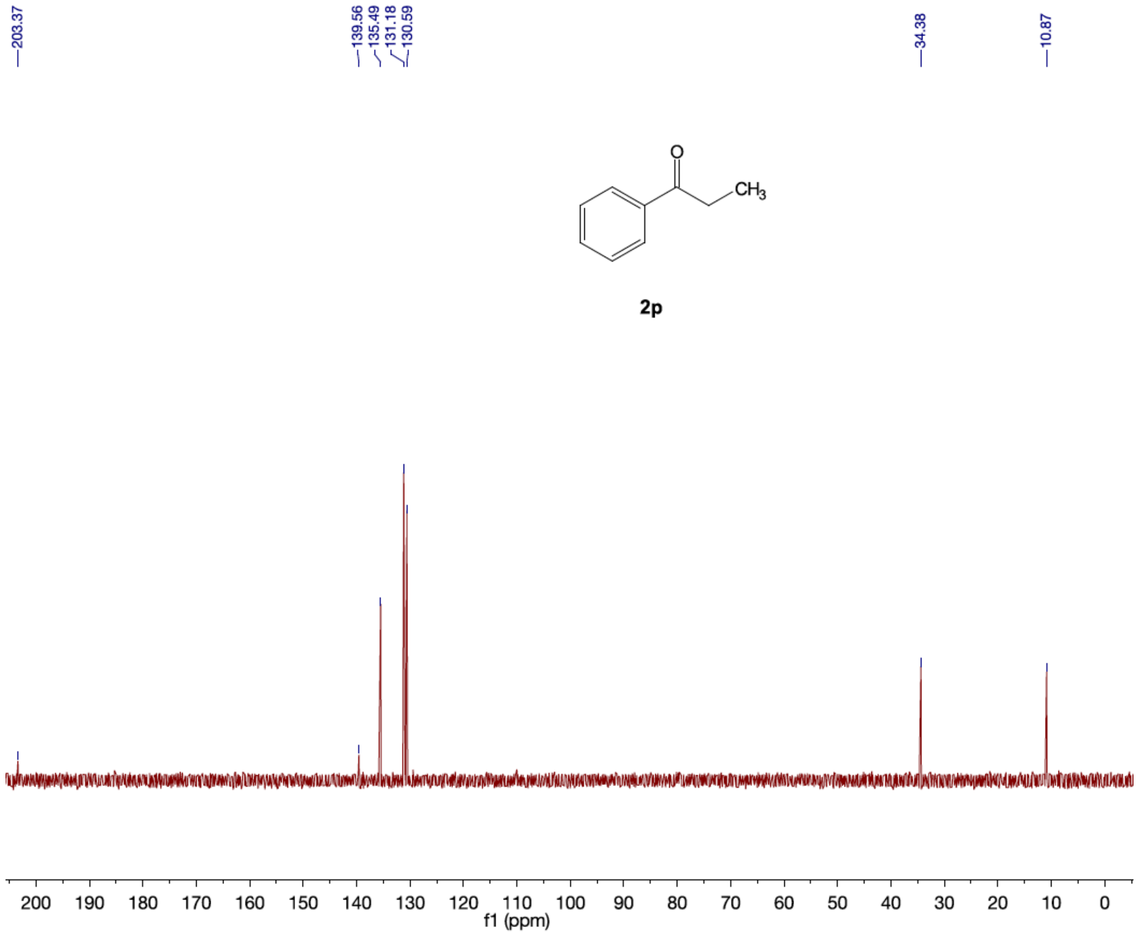
203.37
139.56
135.49
131.18
130.59
34.38
10.87
O
CH3
2p
200 190 180 170 160 150 140 130 120 110 100 90 80 70 60 50 40 30 20 10 0
f1 (ppm)

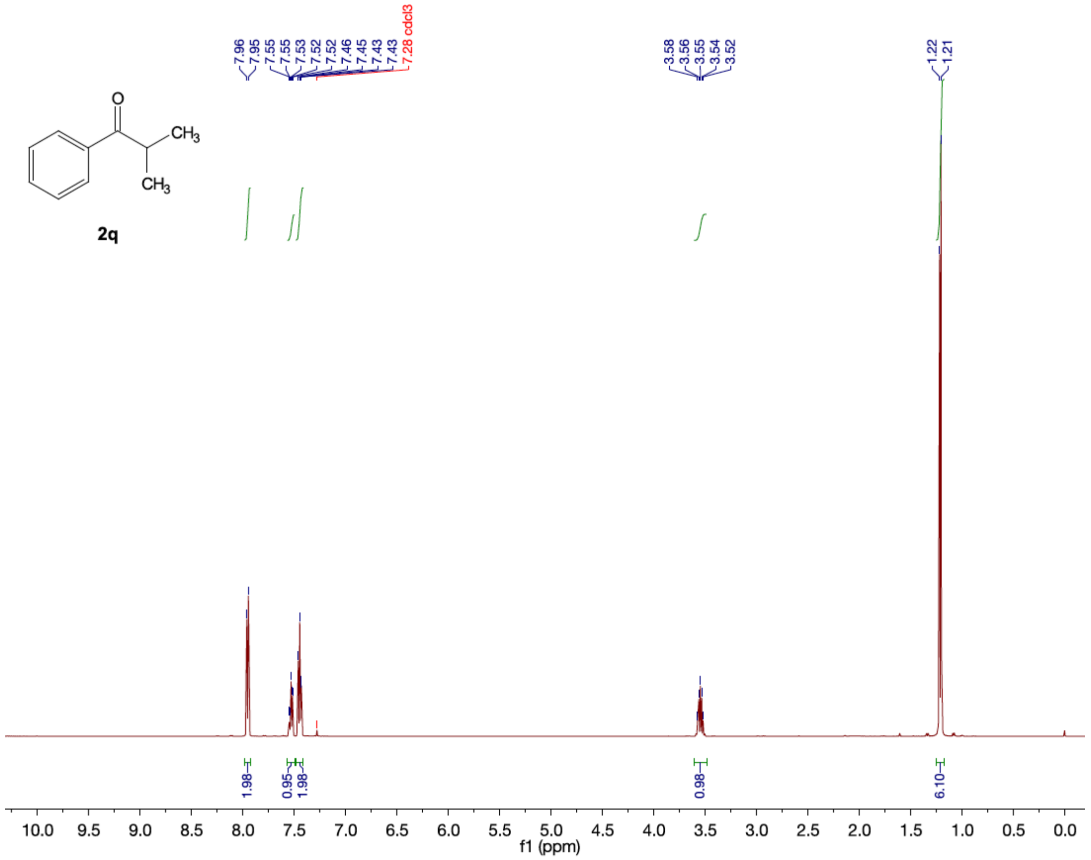
7.96
7.95
7.55
7.55
7.53
7.52
7.52
7.46
7.45
7.43
7.43
7.28 cdcl3
3.58
3.56
3.55
3.54
3.52
1.22
1.21
O
CH3
CH3
2q
1.98
0.95
1.98
0.98
6.10
f1 (ppm)

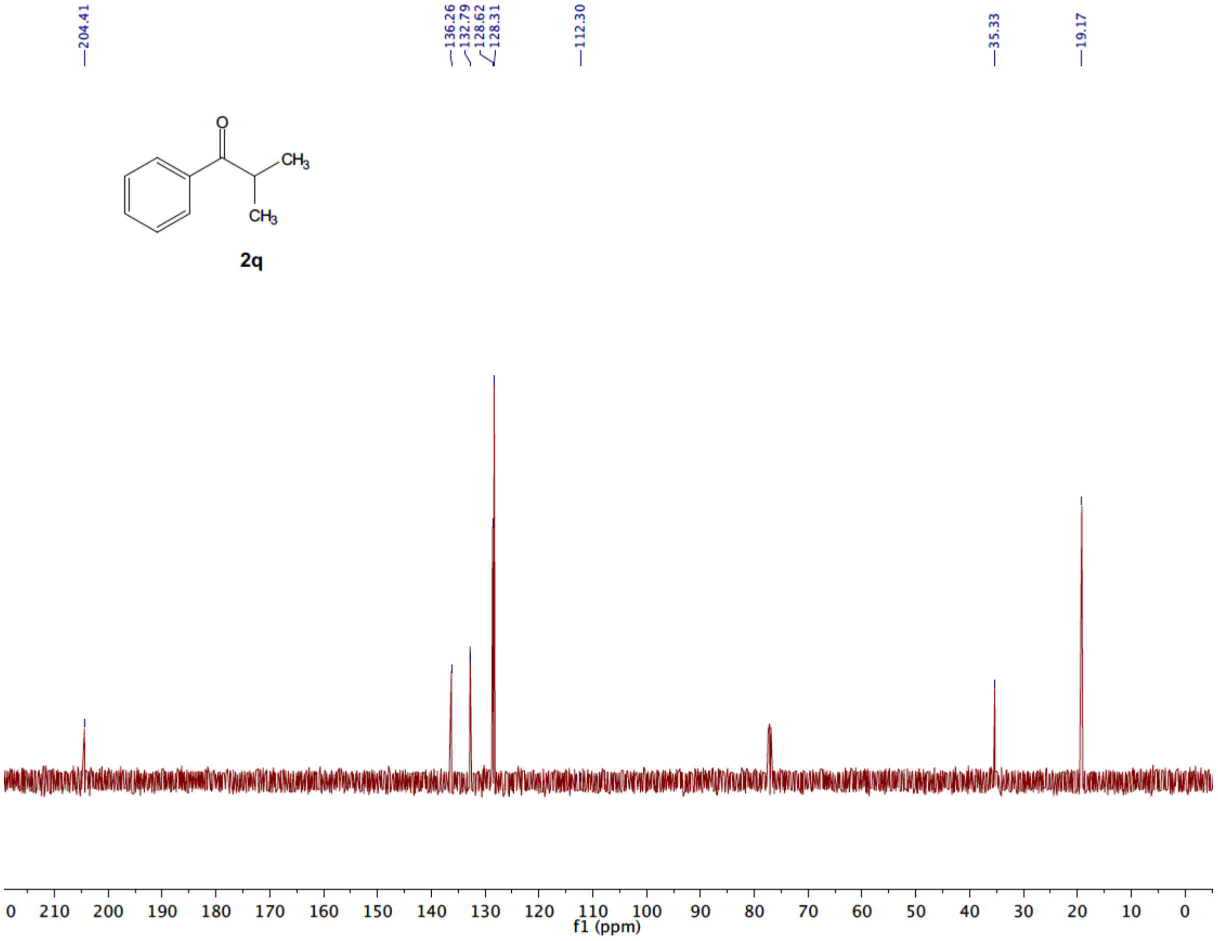
204.41
136.26
132.79
128.62
128.31
112.30
35.33
19.17
O
CH3
CH3
2q
f1 (ppm)

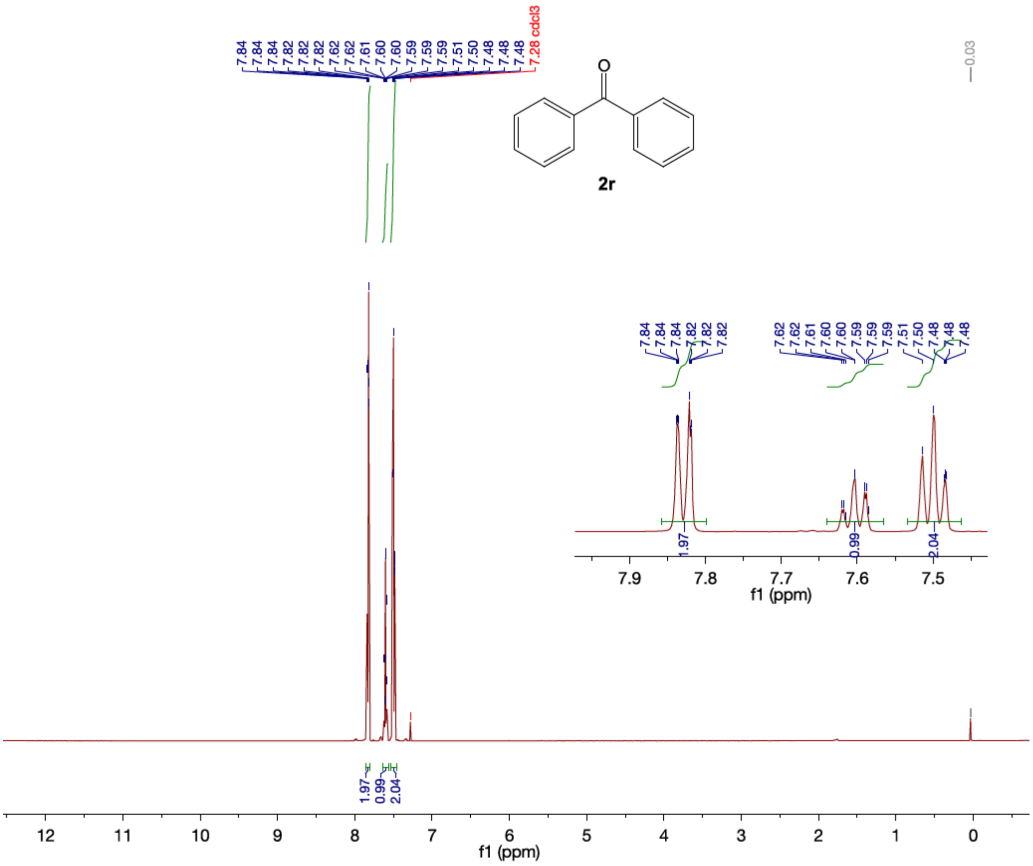
7.84
7.84
7.84
7.82
7.82
7.82
7.62
7.62
7.61
7.60
7.60
7.59
7.59
7.59
7.51
7.50
7.48
7.48
7.48
7.28 cdcl3
—0.03
O
2r
1.97
0.99
2.04
7.9
7.8
7.7
7.6
7.5
f1 (ppm)
12
11
10
9
8
7
6
5
4
3
2
1
0
f1 (ppm)

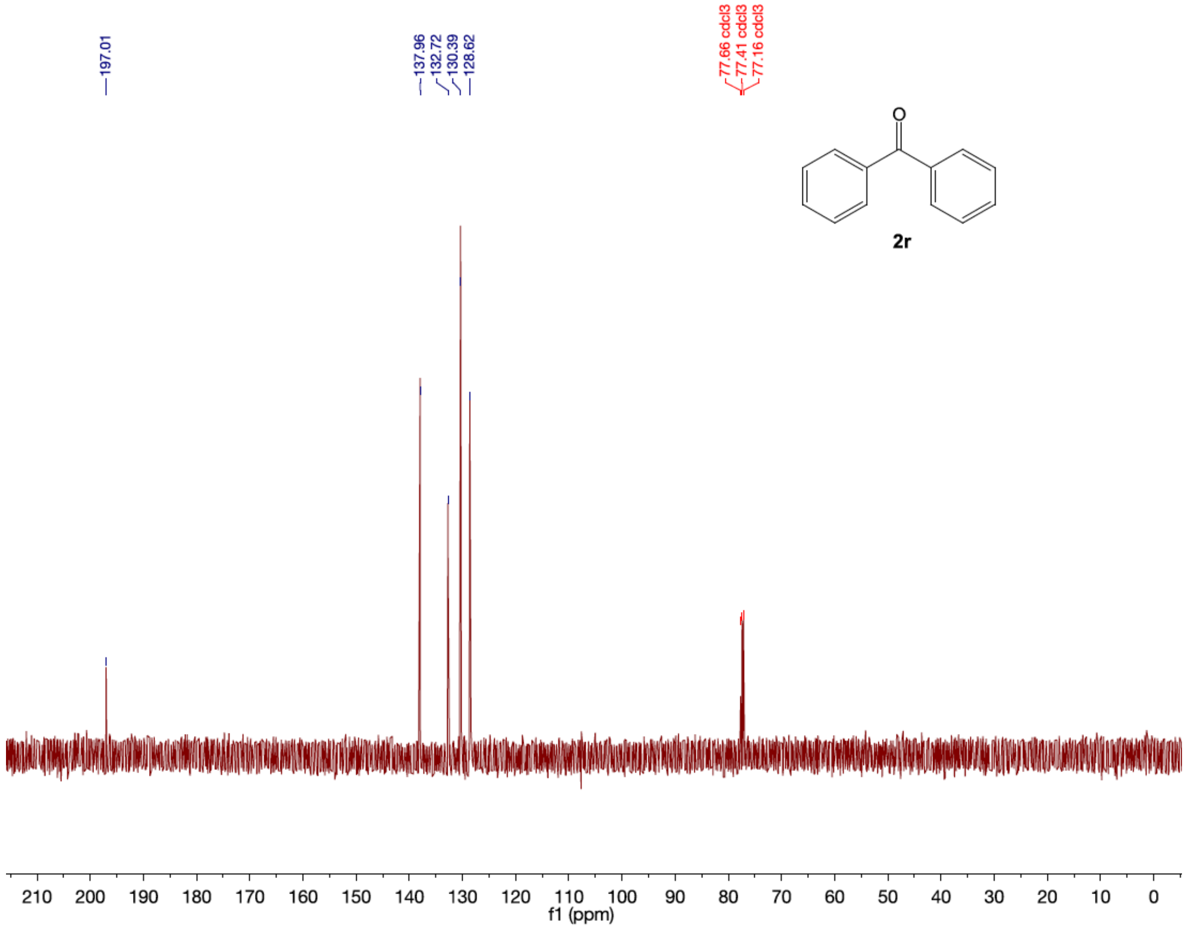
—197.01
137.96
132.72
130.39
128.62
77.66 cdcl3
77.41 cdcl3
77.16 cdcl3
O
2r
210
200
190
180
170
160
150
140
130
120
110
100
90
80
70
60
50
40
30
20
10
0
f1 (ppm)

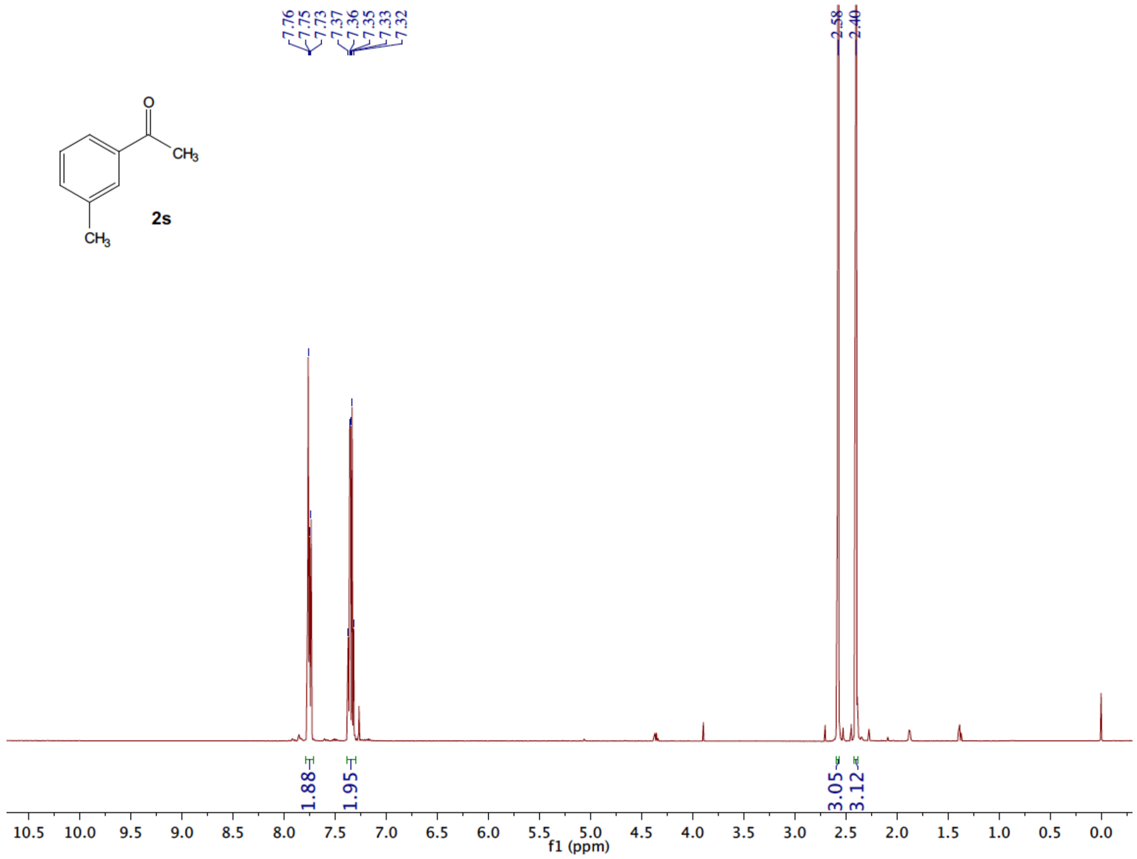
7.76
7.75
7.73
7.37
7.36
7.35
7.33
7.32
2.58
2.40
O
CH3
CH3
2s
1.88
1.95
3.05
3.12
10.5 10.0 9.5 9.0 8.5 8.0 7.5 7.0 6.5 6.0 5.5 5.0 4.5 4.0 3.5 3.0 2.5 2.0 1.5 1.0 0.5 0.0
f1 (ppm)

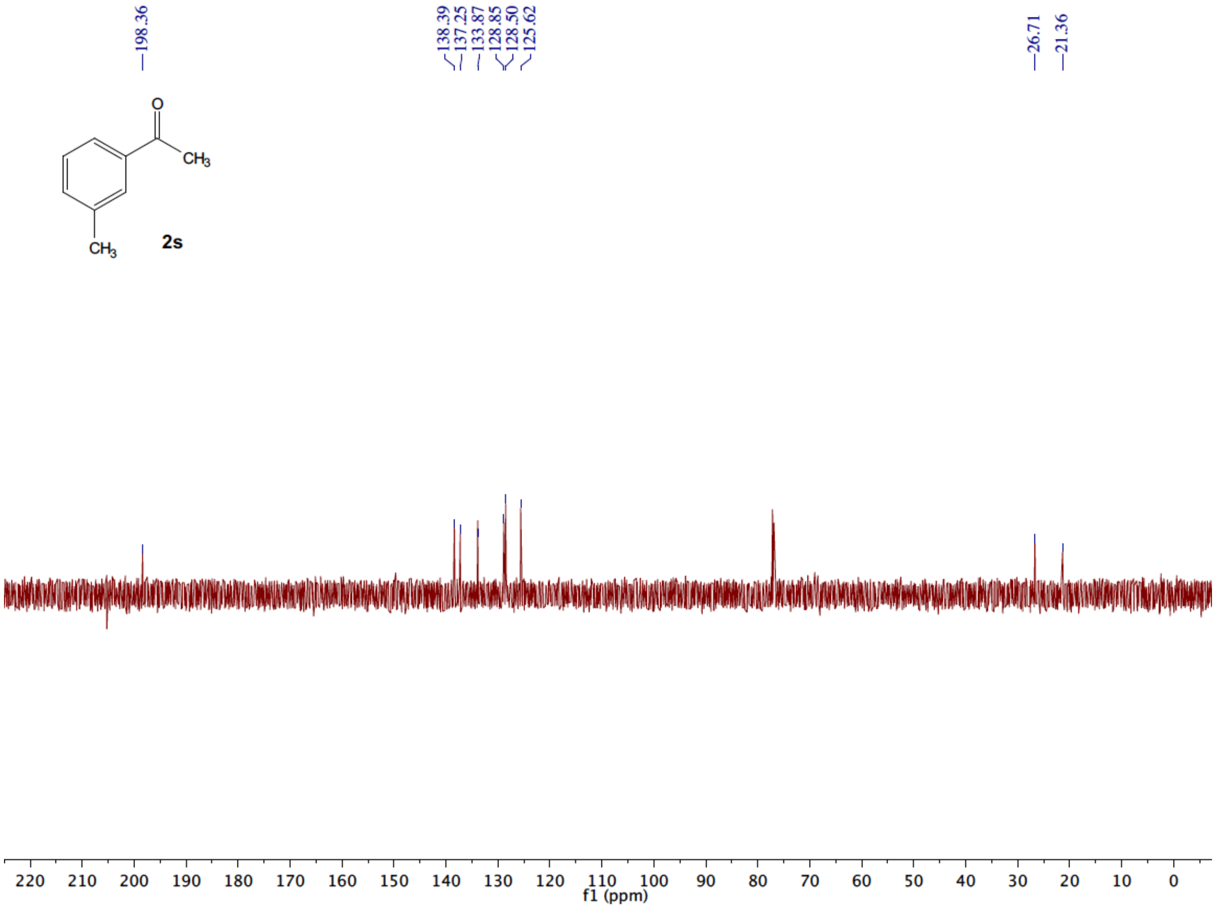
198.36
138.39
137.25
133.87
128.85
128.50
125.62
26.71
21.36
O
CH3
CH3
2s
220 210 200 190 180 170 160 150 140 130 120 110 100 90 80 70 60 50 40 30 20 10 0
f1 (ppm)

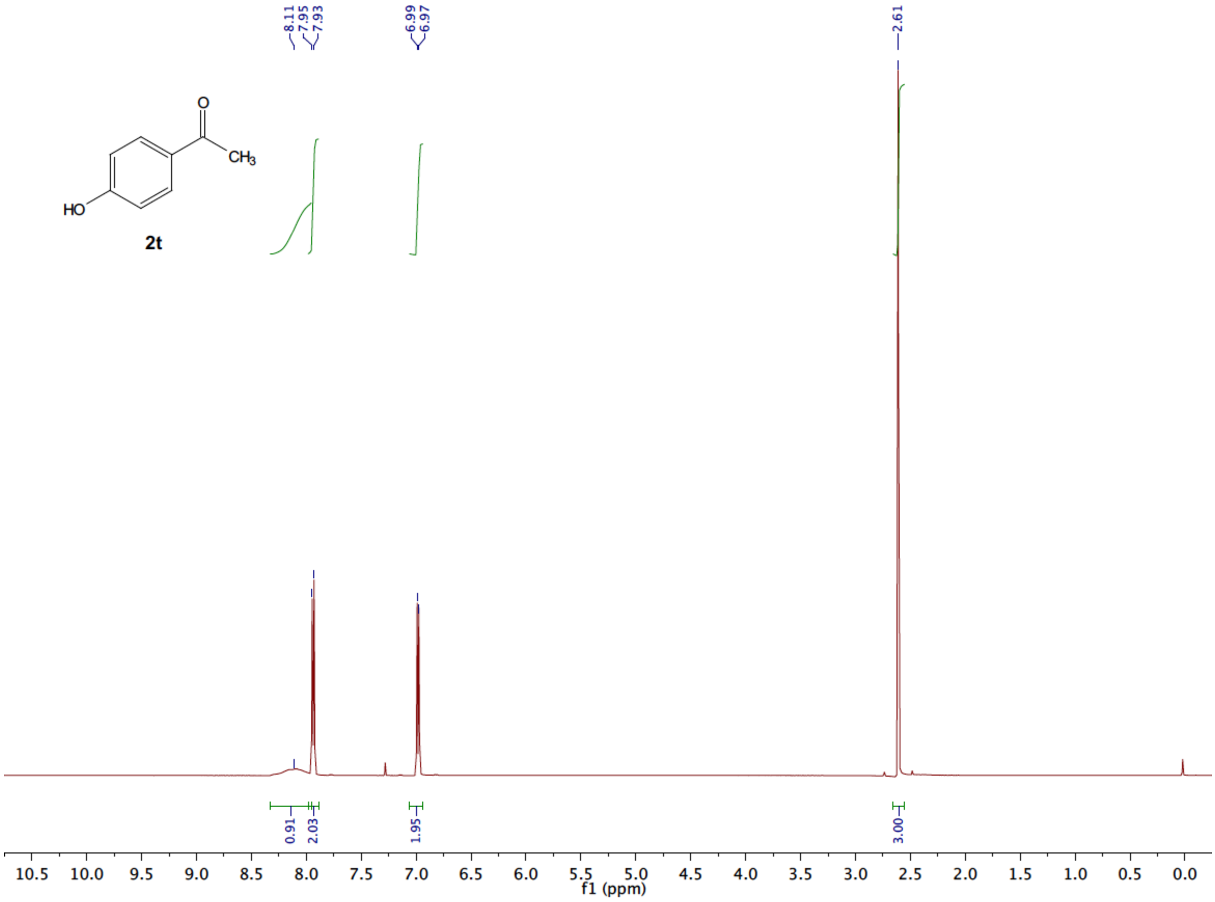

8.11
7.95
7.93
6.99
6.97
2.61
O
CH3
HO
2t
0.91
2.03
1.95
3.00
10.5 10.0 9.5 9.0 8.5 8.0 7.5 7.0 6.5 6.0 5.5 5.0 4.5 4.0 3.5 3.0 2.5 2.0 1.5 1.0 0.5 0.0
f1 (ppm)

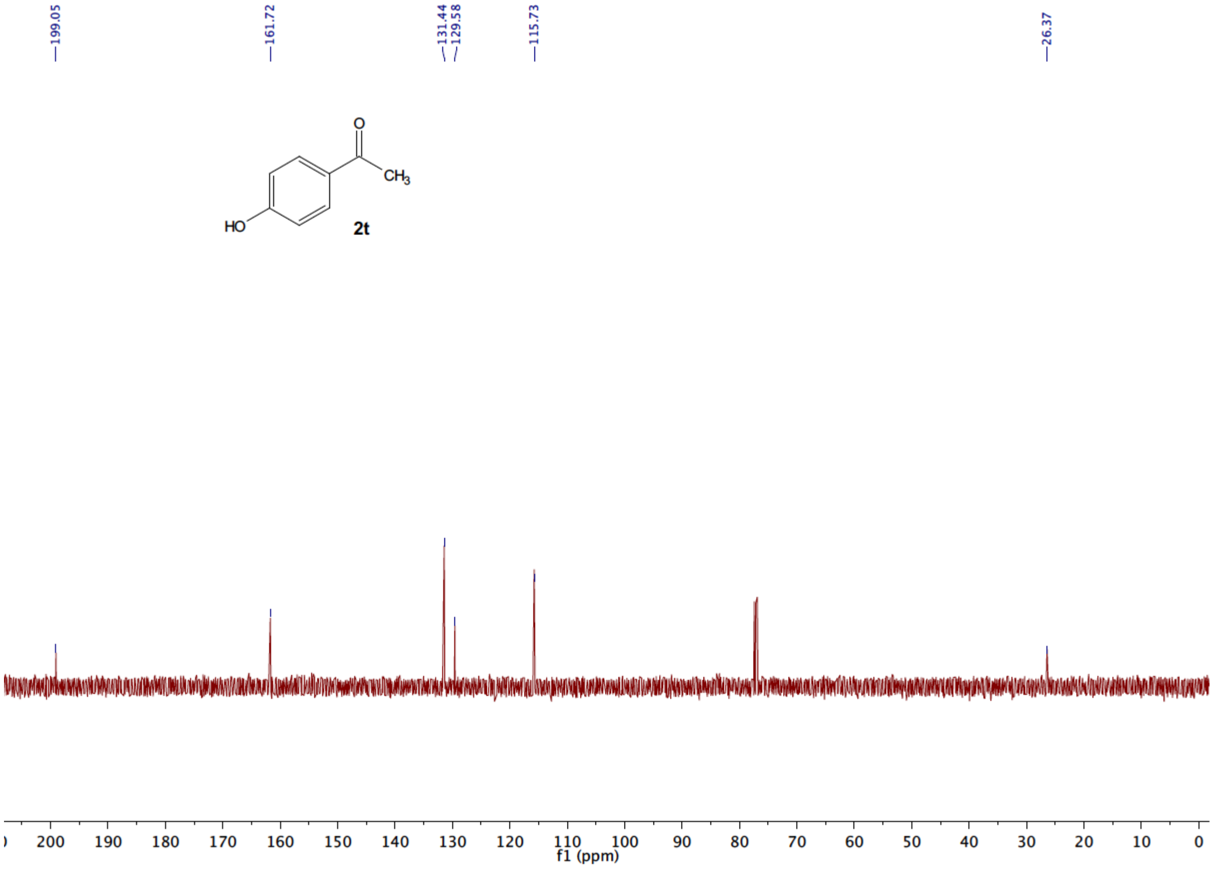

199.05
161.72
131.44
129.58
115.73
26.37
O
CH3
HO
2t
200 190 180 170 160 150 140 130 120 110 100 90 80 70 60 50 40 30 20 10 0
f1 (ppm)

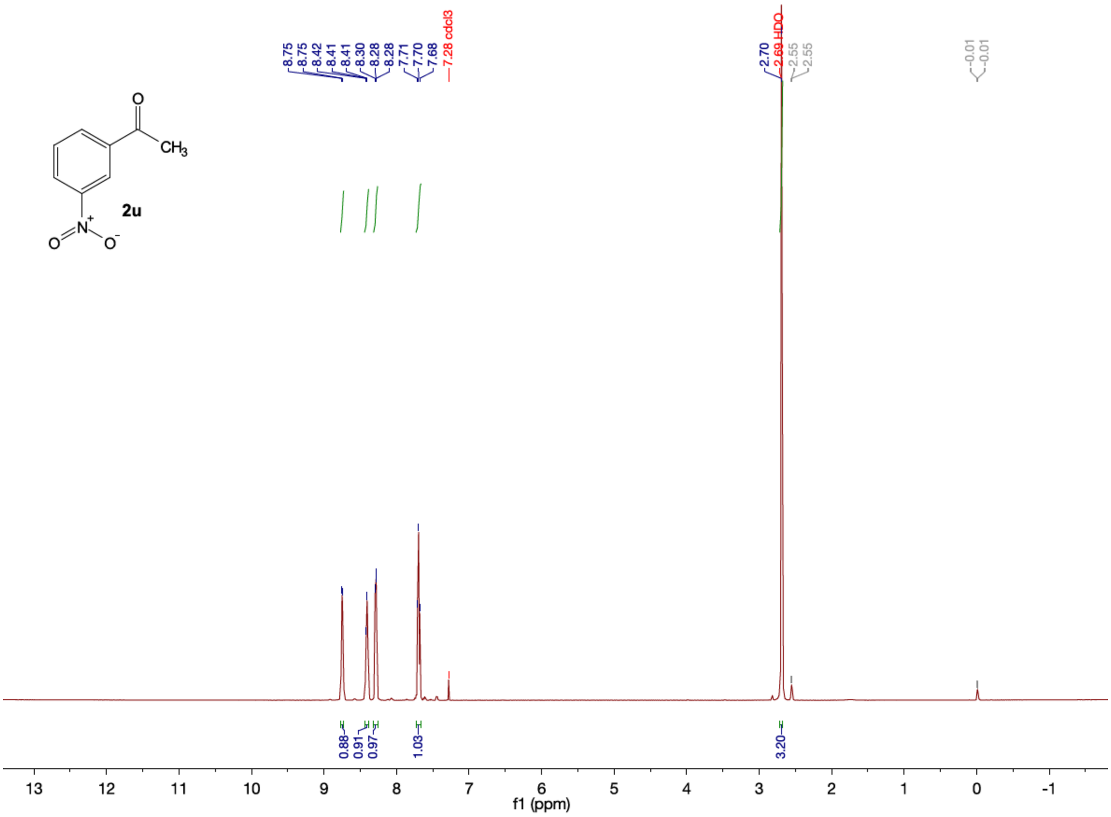
8.75
8.75
8.42
8.41
8.41
8.30
8.28
8.28
7.71
7.70
7.68
7.28 cdcl3
2.70
2.69 HDO
2.55
2.55
-0.01
-0.01
CH3
2u
0.88
0.91
0.97
1.03
3.20
f1 (ppm)

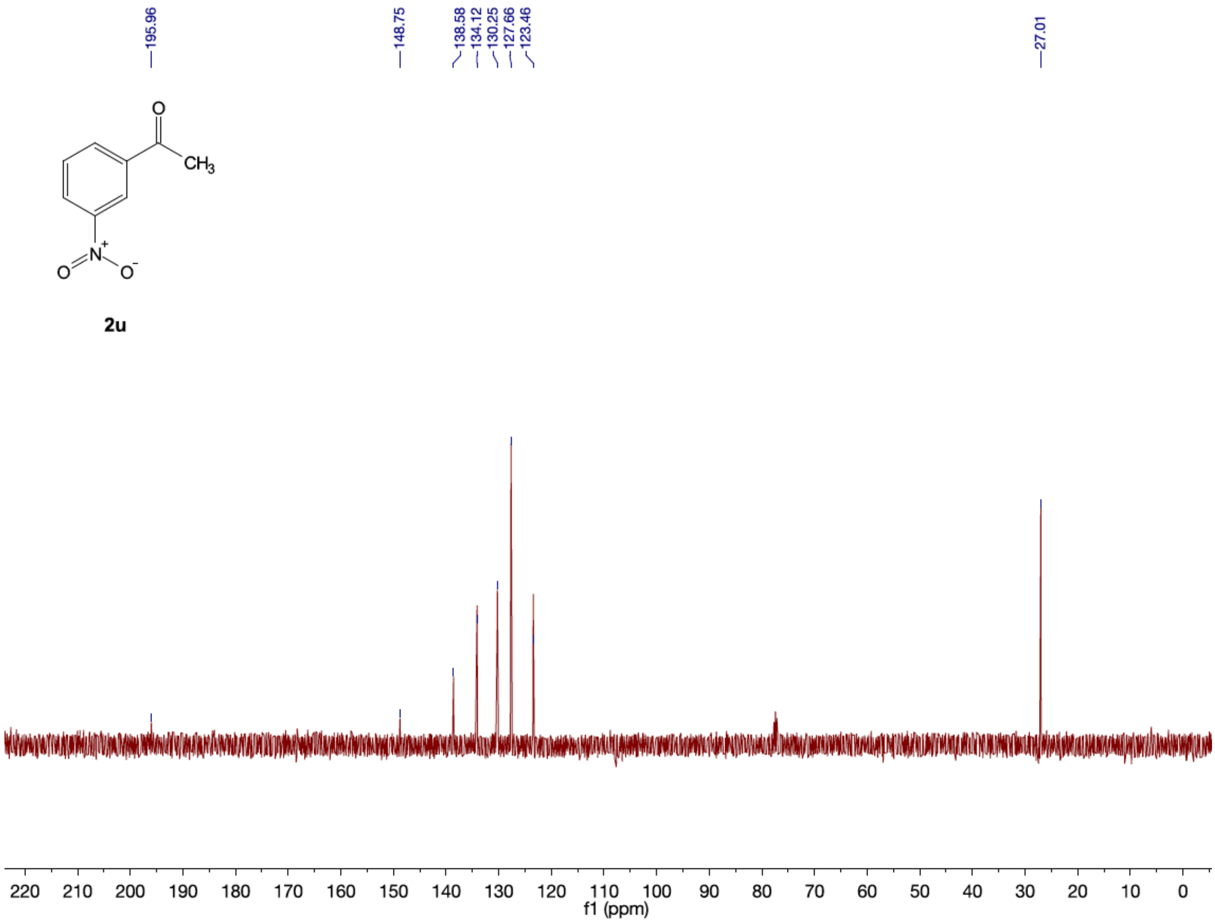
195.96
148.75
138.58
134.12
130.25
127.66
123.46
27.01
CH3
2u
f1 (ppm)

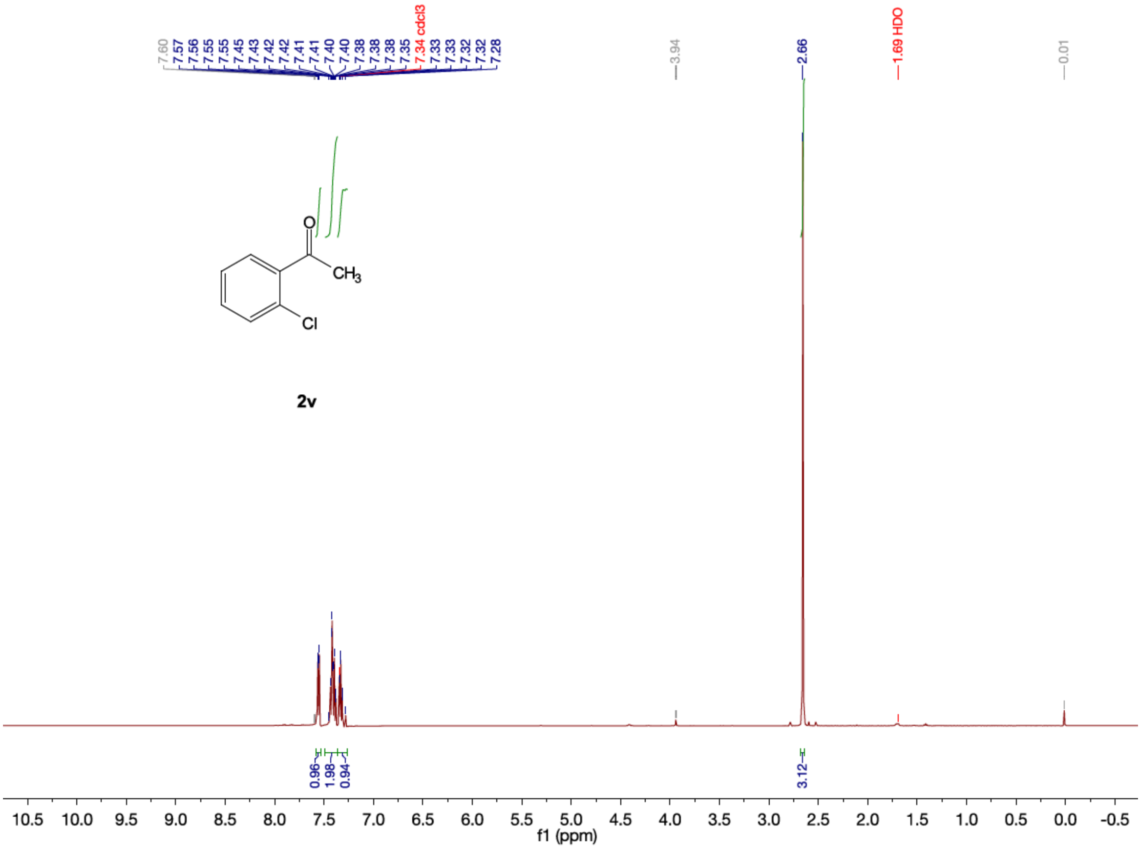
2v

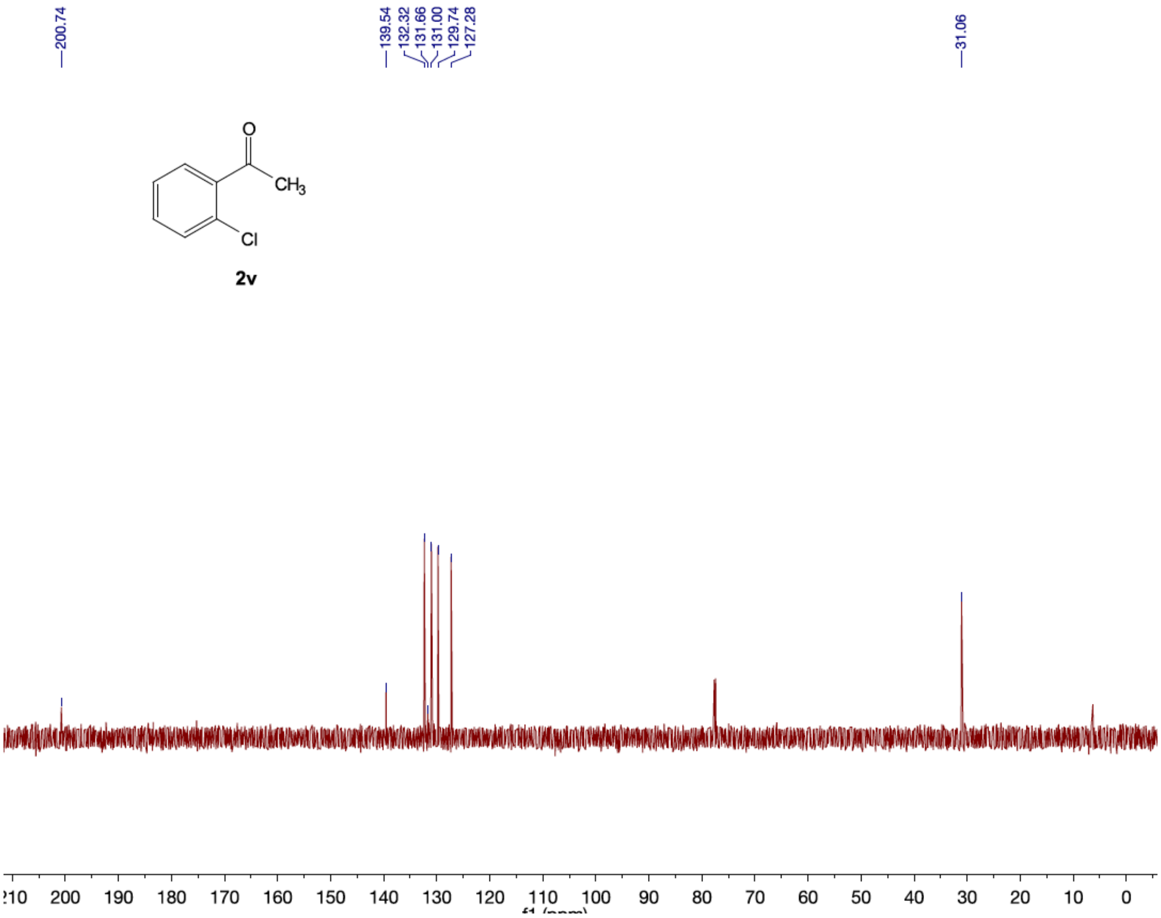
2v
